# Culling a Self-Assembled Quantum Dot as a Single-Photon Source Using X-ray Microscopy

**DOI:** 10.1021/acsnano.3c04835

**Published:** 2023-07-31

**Authors:** Arka Bikash Dey, Milan K. Sanyal, Andreas Schropp, Silvio Achilles, Thomas F. Keller, Ian Farrer, David A. Ritchie, Florian Bertram, Christian G. Schroer, Oliver H. Seeck

**Affiliations:** † Deutsches Elektronen-Synchrotron DESY, Notkestraße 85, 22607 Hamburg, Germany; ‡ Surface Physics and Material Science Division, Saha Institute of Nuclear Physics, Kolkata, West Bengal 700064, India; § Center for X-ray and Nano Science CXNS, Deutsches Elektronen-Synchrotron DESY, Notkestraße 85, Hamburg 22607, Germany; ∥ Physics Department, University of Hamburg, Hamburg 20355, Germany; ⊥ Department of Electronic and Electrical Engineering, University of Sheffield, Mappin Street, Sheffield S1 3JD, United Kingdom; # Cavendish Laboratory, University of Cambridge, J. J. Thomson Avenue, Cambridge CB3 0HE, United Kingdom

**Keywords:** epitaxially grown quantum dots, single quantum dot, single-photon sources, scanning X-ray diffraction microscopy (SXDM), X-ray fluorescence (XRF), compositional inhomogeneities, nanoscale chirality

## Abstract

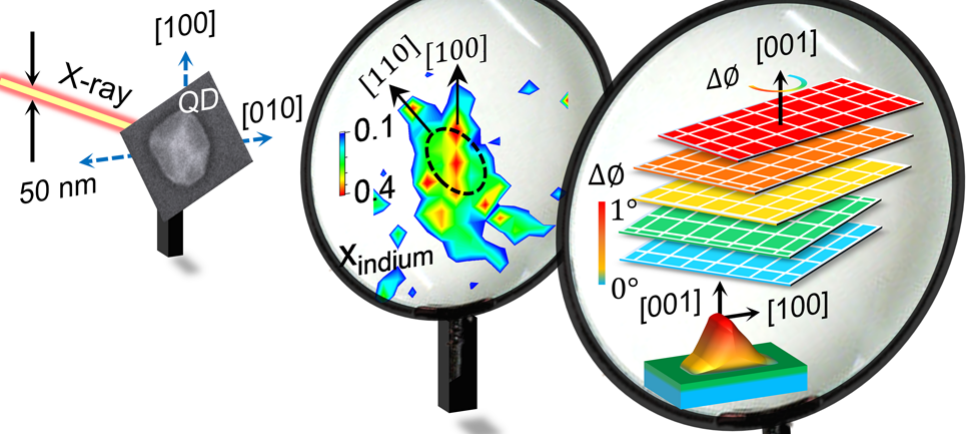

Epitaxially grown self-assembled semiconductor quantum dots (QDs) with atom-like optical properties have emerged as the best choice for single-photon sources required for the development of quantum technology and quantum networks. Nondestructive selection of a single QD having desired structural, compositional, and optical characteristics is essential to obtain noise-free, fully indistinguishable single or entangled photons from single-photon emitters. Here, we show that the structural orientations and local compositional inhomogeneities within a single QD and the surrounding wet layer can be probed in a screening fashion by scanning X-ray diffraction microscopy and X-ray fluorescence with a few tens of nanometers-sized synchrotron radiation beam. The presented measurement protocol can be used to cull the best single QD from the enormous number of self-assembled dots grown simultaneously. The obtained results show that the elemental composition and resultant strain profiles of a QD are sensitive to in-plane crystallographic directions. We also observe that lattice expansion after a certain composition-limit introduces shear strain within a QD, enabling the possibility of controlled chiral-QD formation. Nanoscale chirality and compositional anisotropy, contradictory to common assumptions, need to be incorporated into existing theoretical models to predict the optical properties of single-photon sources and to further tune the epitaxial growth process of self-assembled quantum structures.

Atom-like optical properties^1,2^ of a quantum dot (QD) make them an active source of single^3−5^ or entangled photons^6−8^ required for advanced quantum technologies^9−11^ and communications.^12^ A semiconductor QD-based “single-photon emitter” coupled with nanostructured waveguides^13,14^ or cavities^15^ can produce a single indistinguishable^16^ photon at a time with a high repetition rate^9^ for the development of fascinating quantum technology.^3,4,9,17−19^ The QD-based photon emitters^10^ can provide an entangled photon–exciton state^20^ in future quantum logic operations,^21^ and they are also suitable to investigate the intriguing solid-state quantum electrodynamics that involves light–matter interaction at the most fundamental level.^3,16,22^ Pulsed resonant excitation of the selected single QD is required in these emitters to minimize decoherence^23^ and noise^24^ in single-photon pulses.^5,25^ However, culling of the suitable QD from an enormous number of self-assembled epitaxially grown QDs^26^ is now being carried out only based on optical spectroscopy measurements,^4,27,28^ as a nondestructive way of compositional and structural analysis of a single QD was not possible.

The Stranski–Krastanov (SK) growth mode of epitaxially grown self-assembled QDs is popular because of its defect-free nature.^26^ As they are grown by an ultra-high-vacuum molecular beam epitaxy (MBE) chamber, they are free of any chemical passivation.^1,26^ Therefore, dot size, shape, and aspect ratio, composition and compositional uniformity, crystal quality (including interface quality), and lattice orientation are the most important factors for shaping the optical quality of these self-assembled SK-QDs.^1^ The composition and strain distribution of the materials within a single quantum dot can greatly impact the bandgap and shape of the bandgap, and therefore its optical properties.^29^ Although having identical geometrical sizes and shapes, two SK-QDs can have completely different compositions and strain distributions.^1,26,29^ The homogeneity of the composition and strain within a single QD is one of the key factors for having a sharp (largely reduced full-width half-maxima) optical output.^30^ Another important aspect is crystal quality, which includes the quality of the interface between the QD and the wet layer, which can be understood by the strain at the interface.^1^ The orientation of the QD crystal with respect to the substrate is another important aspect. Therefore, we have focused on the crystallographic orientation, composition and compositional distribution, strain distribution, size, and shape of the studied single QD.

Combining destructive microscopic techniques such as high resolution cross-sectional transmission electron microscopy (HRXTEM)^31^ or scanning tunneling microscopy (STM)^32^ with optical measurements to select the best quality QD is incredibly challenging to implement.^1,33,34^ The composition map of an uncapped QD was determined by an iterative process of elastic energy minimization by simulating composition-dependent lattices in the framework of linear elasticity theory with the help of XTEM measured [001] components of the local lattices and finite elemental method (FEM) by assuming a fixed QD’s shape.^35^ The composition was also evaluated by lattice fringe analysis (CELFA)^33^ with an assumption that all the QDs are identical, random slices of different QDs were linked as if they are positioned at different distances from the center of the QD.^34^ However, this assumption is not true, as QDs are different from one another and there are indeed different sizes of QDs present in single growth condition in SK growth mode.^1^ The HRXSTM and HRXTEM analyses are limited to the structural analysis of a single QD due to three major technical constraints. First, the techniques are limited to the usually small size of the investigated regions. This limits the possibility of measuring the structures of several single QDs in a screening fashion. Second, the size of the QDs is comparable with the thickness of the prepared sample. We found that the observable lateral size by XTEM techniques of the QD appears to be smaller than the actual base size of the QD when the prepared cross-sectional slice is not at the center cross-section of the QD.^30^ Therefore, it is necessary to understand where the prepared slice lies, with respect to the QD’s center. Third, a complicated interpretation of the observed results is needed with extensive modeling, as the contrast depends essentially on both strain and composition, two correlated variables, which results in the observed lattice.^1^

X-ray-based methods are generic tools for investigating the structure of QDs. However, coherent X-ray diffraction (CXD)^36^ cannot provide a satisfactory signal in the lattice/strain map of a single-crystal epitaxial single QD due to the signal overlap of the crystal truncation rod and the Bragg peak^37^ of the QD. Grazing incidence small-angle X-ray scattering (GISAXS) with coherent beam generates the reconstructed average size and shape from the data collected over an ensemble of periodic QDs.^38^ These non-self-assembled QDs^38^ do not have the desired quality of being a “single-photon emitter” due to contaminations and dislocations.^20^ Similarly, grazing incidence diffraction (GID)^29,39^ and X-ray standing wave (XSW)^30^ measure the average structural and compositional information on an ensemble of QDs. X-ray ptychography^40^ can probe the structure and shape of a single nanoparticle.

Here, we demonstrate a protocol based on X-ray microscopy, which is not limited by any of the above-said constraints in different techniques and can easily determine the structure without destroying the QD. Utilizing our protocol, one can measure several single QDs in a screening fashion. Here, we show that the local chemical composition and lattice-strain map of a single QD can be determined through simultaneous measurement of scanning X-ray diffraction microscopy (SXDM)^41^ and elemental X-ray fluorescence (XRF)^42^ with a 30 nm step size with a 50 nm^43^ (refer to “Nano-Focused X-ray Beams” in Section I, Supporting Information (SI)) monochromatic synchrotron X-ray beam in transmission scattering geometry. This measurement protocol can easily locate a single QD through fluorescence mapping to select a suitable QD for developing a single-photon source. No special sample preparation^31,32^ or complex data analysis tools^31,36−38^ (such as a finite element or phase retrieval) are essential here due to separately measuring the composition and strain.^1,29^ The main objective of this paper is to show that a nondestructive X-ray microscopy protocol can determine the complete structure and crystallographic orientation of any single QD in a screening fashion. Here, we study a few single QDs by X-ray microscopy and then probe the same single QDs by surface electron microscopy and atomic force microscopy to ensure that the proposed protocol works. Such complementary measurements are possible only on uncapped QDs. Although the optical emission is weaker in the studied single QDs, it is necessary to study the structure of an uncapped QD first to establish a platform for structural determination of several single QDs in a screening fashion. This protocol is also applicable to detect capped QDs in similar elemental fluorescence mapping, particularly for GaAs capping. A structure correlation between uncapped and capped QDs has been already established earlier.^29^

Before discussing the obtained results, we present a summary of the measurement protocol used in this study; the details of the protocol can be found in Section I in the SI. The protocol is designed for simultaneous measurements of the X-ray diffraction (XRD) and fluorescence (XRF) data from a sample in transmission geometry using a synchrotron X-ray beam. The monochromatic X-ray beams should be focused to nanometer-sized dimensions, smaller than the nano-object being investigated.^43−45^ The sample is mounted on a 3D interferometer feedback piezo stage^43^ and properly oriented to collect diffraction data in the transmission geometry. An area X-ray detector must be positioned on a translational and rotational stage to measure Bragg peaks in the transmission geometry. The substrate thickness should be small to make sure that the diffraction data from the substrate do not overshadow the diffraction data of the nano-object. The energy of the X-ray beam should be higher than that of all elemental Kα or Lα absorption edges of the sample. An energy dispersive X-ray detector is used to collect XRF data of all of the elements present in the sample during a scan. The data treatment for generating SXDM, lattice mapping, and composition mapping is discussed in Section II, SI. By following this protocol, valuable insights into lattice structures, elemental composition, strain distribution, and lattice twists in single nano-objects can be obtained in a screening fashion.

In the present study, an enhanced ratio of the QD diffraction signal to that of the substrate was ensured by reducing the substrate thickness to 40 μm (refer to Figure 1a) (refer to “Sample Preparation” in Section I, SI). Scanning electron microscopy (SEM) and atomic force microscopy (AFM) measurements were performed before (Figure S1c) and after (Figure S1d) the sample preparation process following the measurement protocol step A2 in the SI. Three platinum markers were deposited by ion beam induced deposition (IBID) of a platinum-containing precursor using a focused ion beam (FIB),^46^ two markers at the two edges of the sample (see Figure S2, SI). Another smaller-sized marker was deposited at an approximately 100 μm distance from the QDs for guidance during actual measurement at the synchrotron beamline following measurement protocol step A3 in the SI. Figure 1a represents the schematic of the experimental setup on the ptychographic nano-analytical microscope (PtyNAMi)^43−45^ at the synchrotron radiation beamline P06 at PETRA-III, DESY, which was used for these measurements. A photograph of the experimental setup is shown in Figure S3, SI. The sample was mounted perpendicular to the X-ray beam, with the back side of the substrate facing the incoming beam in transmission geometry as mentioned above. Alignments of the QD, tracking of the precise sample movements, and stabilization of the sample against any unwanted drift was managed using feedback from a three-dimensional interferometric setup at the sample-piezo-stage^43^ (see Figure S3, SI). A photon energy of 28150 eV was chosen to detect Kα fluorescence of indium, which is the highest atomic number in the sample. Higher X-ray energy also provides higher transmission in this geometry. A higher spatial resolution is also achieved due to the lower rotation of the sample (due to lower Bragg angle at higher energy) even at the (400) Bragg condition. The X-ray beam is focused in a beam size of 46 and 49 nm in the vertical and horizontal direction (see Figure S5, SI) with two cylindrical Si lenses integrated into the nanoprobe instrument at the P06 beamline at PETRA III (PtyNAMI),^43^ and the 2D profile of the nanofocused X-ray beam is characterized (see “Nano-Focused X-ray Beams” in Section I, SI) by scanning coherent X-ray microscopy (ptychography) on a Siemens star^43^ using a 2D diffraction detector (Eiger 4M, Dectris Ltd.) following measurement protocol step B6 in the SI. An 2M GaAs lambda 2D detector (XSpectrum) was positioned as shown in Figure 1a (step B7 in the SI) and calibrated with a LaB_6_ standard sample following the measurement protocol step B8 in the SI as shown in Figure S7a. The three platinum markers and the preselected region of interest are relocated by utilizing a guiding marker-based correlative microscopy approach and an in-line optical microscope at the X-ray beamline in a first, rough alignment step following the measurement protocol step B9 in the SI. At beamline P06, the sample alignment was achieved by observing the (400) and (600) Bragg peaks of the substrate, as depicted in Figure S7c. It displays a merged diffraction image captured at Bragg angles of 8.96° for the (400) peak and 13.52° for the (600) peak of the substrate for the X-ray energy of 28150 eV with corresponding *q*-values of 4.45 and 6.67 Å^–1^, respectively. Any sample tilt is compensated by a tilt stage with a ±5° tilt angle. In Figure S7c, the observed joining line between the (400) and (600) substrate Bragg peaks is perfectly horizontal on the detector panel, indicating accurate sample alignment following the measurement protocol step B10 in the SI. An energy-dispersive X-ray detector (Vortex EM with 2 mm SDD, Hitachi) is positioned by optimizing the fluorescence signal following the measurement protocol step B11 in the SI. Finally, a 2D mesh scan was performed around several single QDs with a 30 nm step size with a 50 nm X-ray beam to capture simultaneous XRD and XRF data at each position during scanning following the measurement protocol step B12 in the SI.

**Figure 1:**
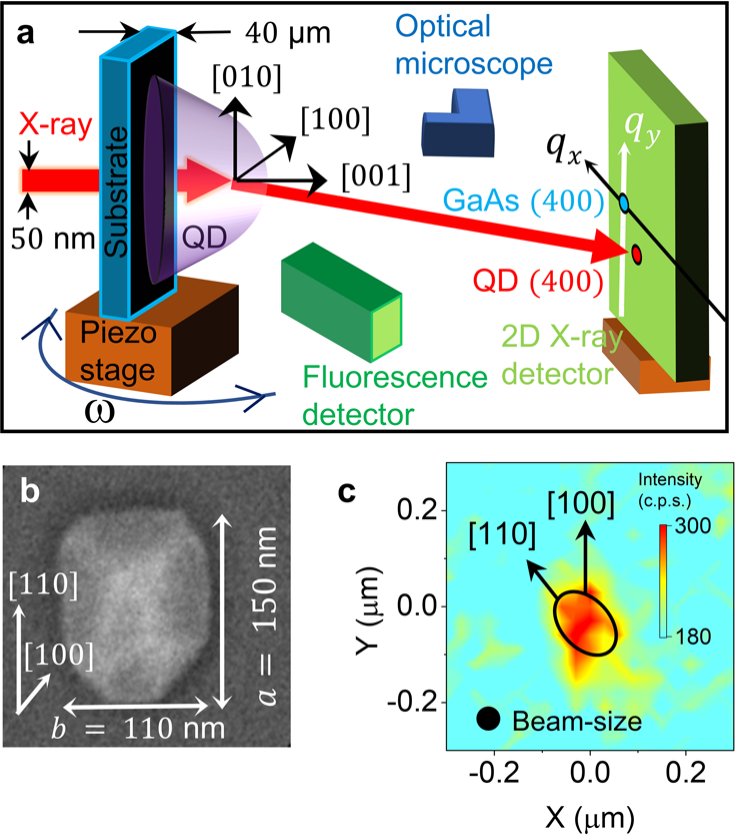
Identifying a single QD in a screening fashion in the transmission scattering geometry. (a) Schematic of the experimental setup used to probe a single QD in a screening fashion. (b) SEM image of the first preselected QD1 shows lateral dimensions of 150 nm × 110 nm. (c) Indium Kα fluorescence intensity mapping of the QD1 during mesh scan around the QD1. The black ellipse represents the position of the QD1. The unit of the color bar is intensity counts per second (c.p.s.).

## Results/Discussion

We have used the self-assembled QDs of indium gallium arsenide (InGaAs) grown on a GaAs (001) substrate using MBE^21,23,26^ (refer to “Sample Growth” in Methods). A variation in sizes and shapes in the ensemble of self-assembled QDs^1,26^ is a common phenomenon as observed in this sample (refer to Figure S1, SI). Here, we discuss the results of two representative QDs, which were characterized by SEM and AFM (refer to Figure S1). The first preselected QD (QD1) has a length of 150 nm, a width of 110 nm (Figure 1b), and a height of 20 nm (refer to Figure S1). The second preselected QD (QD2) has a length of 144 nm, a width of 100 nm, and a height of 20 nm (Figure S10a). The figures corresponding to the QD2 are presented in the SI because the results obtained from the QD2 are similar to the results obtained from the QD1. The QD1 (refer to the black ellipse in Figure 1c), QD2 (refer to the black ellipse in Figure S10b), and their surrounding interface are found and characterized by the indium Kα fluorescence intensity mapping with a 30 nm step size with an X-ray beam size of 50 nm. The single QDs and their interface with the substrate can be seen as red (higher intensity) and yellow (medium intensity), respectively, in the indium XRF intensity map. There is a clear difference between the region occupied by indium atoms (Figure 1c) and the shape of the single QDs (Figure 1b) observed by microscopy measurement. It is to be noted that the XRF map is sensitive to the indium concentration, whereas the SEM and AFM are sensitive to the physical boundary of the QD.

**Figure 2:**
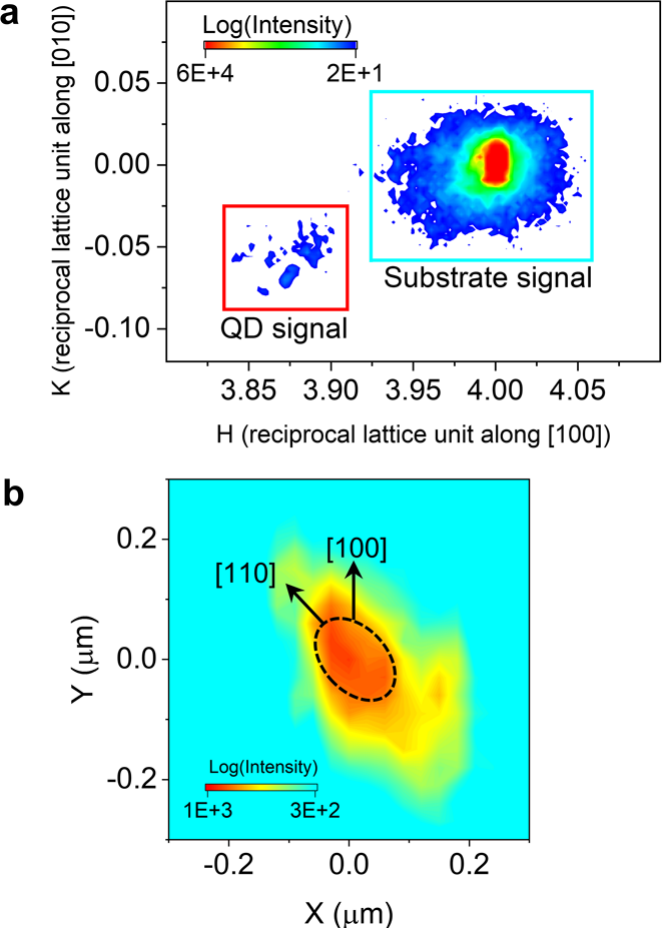
Scanning X-ray diffraction microscopy on the single QDs. (a) Reciprocal space mapping of the (400) diffraction signals when the X-ray beam hits the regions within the QD1 (within the black ellipse in Figure 1c). *H* and *K* in the *x*-axes and *y*-axes, respectively, denote Miller indices of the GaAs substrate. (b) Scanning X-ray diffraction microscopy of the *Q*-space enclosed within the red box in (a). The color bar is in units of intensity counts per unit second and mapped in logarithm (log) scale.

We collected the (400) diffraction data from the aligned QDs by scanning (a typical 2D mesh scan with a 50 nm monochromatic X-ray beam) the sample region enclosed by the black ellipse shown in Figure 1c. Both the diffraction and fluorescence data were collected simultaneously at each position during the 2D mesh scan. Figure 2a shows the reciprocal space mapping around the (400) Bragg peak of QD1 and the GaAs substrate as obtained from the entire mesh scan. The substrate, which is present under the QD1 within the black ellipse (refer to Figure 1c), gives an intense (400) Bragg peak, shown as a blue box in Figure 2a. We observe less intense diffraction peaks at lower *H*-values (the red box in Figure 2a) in reciprocal (*H*–*K*) space coming from the QD1. As the measured diffraction intensities are stored from each position during the mesh scan, one can infer the real-space location on the sample that gives rise to diffraction data, and this is the basis of the SXDM technique. For example, the measured integrated diffraction intensity in the reciprocal space marked by the red box of Figure 2a (a square region on the detector) can be mapped back to the corresponding positions on the 2D mesh scan grid of the real-space positions of QD1. Figure 2b depicts the real-space SXDM image of the integrated diffraction intensity on the red box in Figure 2a. This region encompasses QD1 and its surrounding interface, which are distinctly discernible in the SXDM image. This provides a platform for a deeper understanding of the QD’s internal structure and highlights the capability of SXDM in nanoscale materials. For example, we observed that the in-plane area observed (red center, Figure 2b) by SXDM is slightly larger than the area that contains higher indium (red center, Figure 1c) and the physical boundary of the QD1 (refer to Figure 1b) as measured by microscopy techniques (SEM, AFM). This observation indicates that the lattices at the interface between QD1 and the substrate get affected over larger length scales. For consistency, we presented the results obtained from the QD2: the reciprocal space mapping of (400) diffraction data in Figure S10c in the SI and SXDM in Figure S10d in the SI. Both quantum dots QD1 and QD2 show similar results.

**Figure 3:**
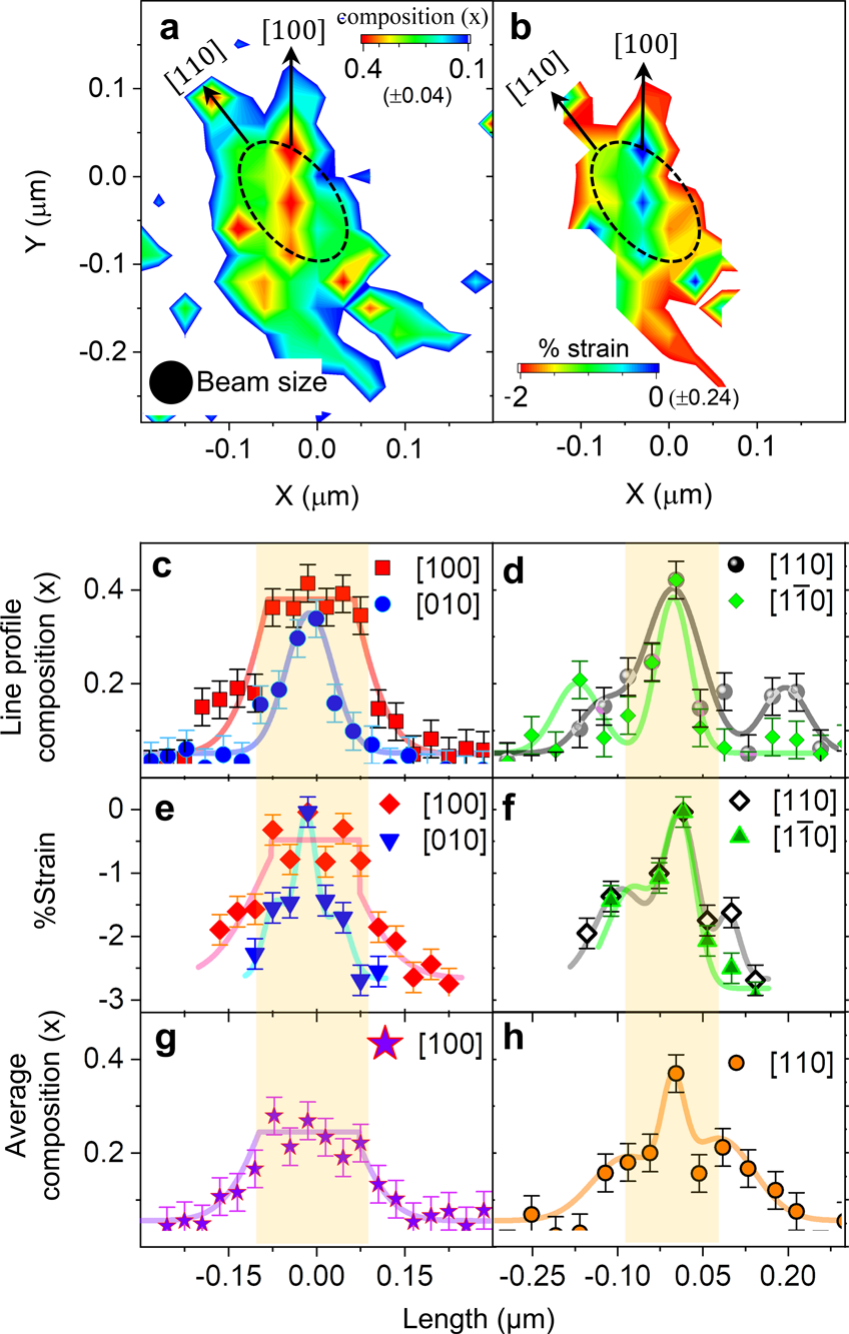
| Directional anisotropy in composition and strain within the QD. (a) Indium composition mapping within the QD1 and its interface as obtained from elemental X-ray fluorescent signals for the QD1. (b) Absolute in-plane strain (in %) mapping within the QD1 and its interface as obtained by comparing the lattices calculated by Vegard’s law from the indium composition in (a) and the lattices measured from X-ray diffraction. (c) Line (∼ 50 nm width) profiles of the indium composition along (c) [100] and [010] and (d) [110] and [11̅0], passing through the center of the QD1. The average indium compositions of the QD1 and its interface along the (e) [100] and (f) [110] directions. The yellow highlighted regions represent the regions inside the QD1. Line (∼50 nm width) profiles of average composition within the QD1 and its interface are plotted along the (g) [100] and (h) [110] directions.

We observe directional anisotropies in the in-plane distribution of composition (Figure 3a) and strain (Figure 3b) within the single QD. The XRF data obtained during SXDM measurement produce an indium composition map (*x*) (refer to “Composition Map”, Methods) within the QD, where the QD material is denoted by In_*x*_Ga_1–*x*_As. The error bar in the indium composition map comes from the standard deviation of the noise. We obtain that the maximum compositional error is ±0.04. The composition map (refer to Figure 3a) shows that the indium loading within the QD is different along the [110] and [100] crystallographic directions. To further illustrate the directional̅ asymmetry in the elemental composition, we show a few indium composition line profiles of about 50 nm width through the center of the QD along [100] and [010] (refer to Figure 3c) and along [110] and [11̅0] (refer to Figure 3d). The yellow highlighted regions in these figures represent the regions inside the QD. We observe an almost constant indium concentration (∼0.4 ± 0.04) within the QD in the line profile along [100]. Few prepared slices of QDs, grown at different temperatures between 480 and 530 °C, are studied by X-TEM and X-STM where a comparatively higher indium concentration is observed.^34,35,47,48^ The heights of these QDs varied between 2.2 and 9.4 nm. Due to the larger size of the studied QD of height around 20 nm compared to the previously studied QDs by X-TEM and X-STM, we observe that the maximum value of indium concentration is lower (*x* ∼ 0.4) than that observed for smaller-sized QDs (*x* ∼ 0.6–0.7). At the interface of the QD, a sharp decrease of indium concentration to ∼0.2 ± 0.04 is observed from the flat-top profile along [100] as it approaches the wetting layer (WL). The WL is the thin 2D layer that is just above the substrate.^1^ In contrast to the indium concentration distribution along [100], the indium concentration is only higher in the central portion of the QD and drops dramatically, giving a “Gaussian-like” profile in other crystallographic directions, [010], [110], and [11̅0]. We determine strain (ϵ) from the differences between measured lattice parameters, *a*_XRD_(*x*), from (400) diffraction data (refer to “Lattice Map”, Methods) and Vegard’s law calculated lattice parameters, *a*_XRF_(*x*), from the obtained composition through the XRF map. In Figure 3b, we show the obtained in-plane strain map of the single QD defined as ϵ_II,abs_ = [*a*_XRD_(*x*) – *a*_XRF_(*x*)]/*a*_XRF_(*x*).^29^ The maximum relative strain error in the strain mapping is ±0.24%. The strain map within QDs shows compressive strain, as expected,^26,49^ except in the areas having maximum indium concentration, where the strain value decreases drastically. The directional anisotropies in the strain show a trend similar to that obtained in the composition map and show a flat compressive strain close to zero (∼−0.2 ± 0.24%). The flat compressive stress along [100] (red line in Figure 3e) becomes more compressive (∼−2 ± 0.24%) toward the interface. Strain profiles in other crystallographic directions, along [010] (blue line in Figure 3e) and along [110] and [11̅0] (black line and green line in Figure 3f, respectively) are quite different (Gaussian-like profile) from that observed in the [100] direction (flat profile) within the QD. The previously reported^29,39^ composition/strain variation was obtained for the average QD through measurements carried out on an ensemble of QDs. HRXTEM measurements^26,32,49,50^ reported earlier on a single QD showed an inhomogeneous indium distribution within the dot along the [001] growth direction. The in-plane indium distributions within the single QD along different directions are difficult in microscopic studies^26,32,49,50^ and are assumed to be independent of in-plane crystallographic directions. The nature of the indium concentration and strain line profiles remains very different in various in-plane directions even after integrating over the entire QD (refer to Figure 3g,h); this lateral anisotropy is a primary observation of this study, and it raises a question regarding the effectiveness of the theoretical models to calculate optical properties that assume lateral azimuthal symmetry.^1,35,47,48,51^ The composition (Figure S11a, SI) and strain (Figure S11b, SI) map of the QD2 show lateral anisotropy, similar to that discussed for QD1. The maximum indium composition also reaches around 0.4 ± 0.04, and strain remains at the minimum value at the center of the QD2.

**Figure 4:**
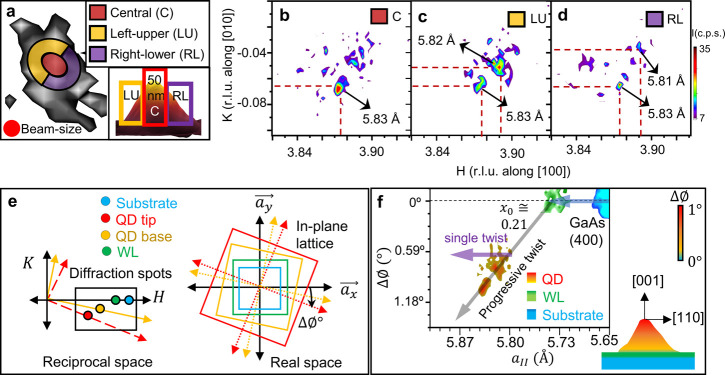
QD’s lattice expansions and chirality. (a) Three different portions (three colors) of the QD (QD1) are shown to demonstrate the (400) diffraction intensities from the different portions. The inset shows the atomic force microscopy (AFM) image to demonstrate how an X-ray hits different portions in a cross-sectional view. (b) Central (C), (c) the left-upper (LU), and (d) the right-lower (RL) portions of the QD1 using the (400) diffraction. (e) A schematic representing how the diffraction spots are related to the in-plane lattice of the QD1, WL, and substrate. (f) Lattice expansions and angular rotation of the QD1 (red-yellow), wet layer (green), and substrate (blue) vary linearly. The schematic shows the vertical positions of the in-plane lattices within the QD1 (in a cross-sectional view).

We identify different portions of the QD1 as central (C), left-upper (LU), and right-lower (RL), which are marked by red, yellow, and violet colors, respectively, in Figure 4a to understand the nature of the lattice distortion. The corresponding (400) diffraction signals of these three portions of QD1 are shown in the reciprocal space (*H*–*K*) map in Figure 4b–d. The central portion of the QD1 (“C” region) contains the QD1 tip (refer to the AFM image in the inset in Figure 4a) and has the maximum indium concentration. The “C” region exhibits mostly a larger lattice (5.83 Å) (Figure 4b) as expected for higher (∼0.4) indium composition (Figure 3a) and has negligible strain (∼−0.02%), as discussed above (Figure 3b). The peripheral regions, “LU” and “RL”, represent the base regions of the QD1. The “LU” and “RL” regions show primarily a lattice constant of 5.82 Å (Figure 4c) and 5.81 Å (Figure 4d), respectively, due to a relatively lower indium content (Figure 3a). The traces of 5.83 Å lattices are also obtained in both positions of QD1. Similarly, the central region of the QD2 contains a higher lattice (5.82 Å) compared to the lattices (5.80 Å) present in the “LU” and “RU” region of the QD2 as shown in Figure S12, SI. The (110) cleaved surface of the previously studied InGaAs QD^32^ showed a similar trend of having a higher lattice (∼6.06 Å) at the center and a lower lattice (5.86 Å) at the side of the QD. All three portions of the QD1 exhibit (400) diffraction signals at nonzero *K*-values (Figure 4b–d), and the *K*-values approach zero as indium concentration decreases to reach the GaAs substrate (400) peak.

The nonzero *K*-values imply that (400) planes of a QD are not parallel to the real-space lattice vector of the substrate in the *y*-direction, *a*_*y⃗*_. Instead, the planes are inclined by some angles (ΔΦ) to *a*_*y⃗*_ as shown in Figure 4e. For consistency throughout Figure 4e,f, the red, yellow, green, and blue colors represent the QD tip, QD base, WL, and substrate, respectively. The rotation angle can be quantified by ΔΦ = tan^–1^(*K*/*H*). The values of the in-plane lattice rotations are found to be ∼1°, 0.58°, and 0° at the QD tip, QD base, and wet layer, respectively, with respect to the substrate in-plane lattice. This in-plane rotation can be understood by in-plane shear stress. The shear strain on a crystal lattice can introduce in-plane lattice rotation by causing a change in the crystal’s orientation. In a planar view, stress has four components: two normal stress components (*σ*_*xx*_, *σ*_*yy*_) and two shear stress components (*τ*_*xy*_, *τ*_*yx*_). A nonzero value for the planar shear stress is responsible for a planar rotation of the lattice. The shear stress originates from the unequal composition of neighboring unit cells in different crystallographic directions. In the case of any shear stress, the rotation angle (ΔΦ) is proportional to the applied shear torque. Here, the torque comes from the anisotropic lattice expansion due to the anisotropic composition distribution of the indium, as shown in Figure 3 and discussed in the previous section. This rotation (ΔΦ) can be quantified by measuring the relative orientation of the crystal lattice planes. The (400) diffraction data of the QD [in red (tip) to yellow (base) gradient color], wet layer (green color), and substrate (blue color) are shown with in-plane lattices and the rotation angles in Figure 4f. We observe two trends; the first trend (blue arrow in Figure 4f) shows only lattice expansion without any shear stress. This is the case for the WL (refer to green signals in Figure 4f). The (400) diffraction signal for the WL comes at a zero *K*-value (ΔΦ = 0° line in Figure 4f), indicating no resultant shear force due to a uniform composition^26^ within the 2D thin layer. The second trend (gray arrow in Figure 4f) shows lattice expansion under shear stress as the indium composition increases inside the QD. The rotation angle varies linearly with the lattice expansion in this region. The tip region (a larger lattice) has a higher in-plane lattice rotation, and the base region (a smaller lattice) has a lower in-plane lattice rotation with respect to the substrate in-plane lattice (Figure 4f). A linear extrapolation of the second trend (gray arrow in Figure 4f) intersects the first trend (*K* = 0 axes in Figure 4f) precisely at the WL in-plane lattice. It leads to two crucial interpretations; first, a QD’s in-plane lattice can only expand without shear stress if the elemental composition (here, indium) stays below a certain value (here, *x*_0_ = 0.21 ± 0.01). The value of *x*_0_ for the QD2 is also around 0.21, as shown in Figure S13b, SI. Second, the wet-layer lattices lie at the intersection point of the two observed trends and act as a mediator between a QD’s lattices and its substrate’s lattices. We interpret that the action of mediator of the WL provides the perfect interfacial platform.

This study demonstrates that the lattices of the QDs exhibit twisting relative to both the substrate and the wet layer. Within the QD structure, two possibilities can occur. The first possibility suggests that the QD lattice maintains a constant twist angle throughout its height while undergoing lattice expansion due to compositional changes from the base region to the apex of the dot. The second possibility suggests a continuous twist throughout the QD’s height, accompanied by lattice expansion, indicating chirality. The X-ray diffraction technique allows us to distinguish between these two possibilities. In the first scenario, the (400) QD Bragg intensity should distribute along any parallel line of the violet arrow in Figure 4f, where rotation angle remained constant in the (ΔΦ, *a*_II_) space for QD1. Conversely, in the second scenario, where a continuous twist is present, the (400) diffraction signal distribution should not follow the violet arrow or any arrow that is parallel to the violet arrow in Figure 4f. For QD1, the (400) diffraction signal follows the gray arrow in Figure 4f, intersecting the violet arrow at a specific angle. This suggests that QD1 falls under the second scenario, where a progressive change in the twist angle of the in-plane lattice is observed. The intersection angle between the violet arrow (representing a specific twist case) and the gray arrow (depicting the actual distribution of the (400) diffraction signal in reciprocal space mapping) provides insights into the rate of change of the twist angle along the height of the QD.

As the indium composition increases from the base toward the tip of the QD, our results suggest progressive rotation of the in-plane lattice to form a helix naturally observed in the chiral nanostructures.^52−55^ Such a progressive in-plane rotation at different heights along the length of a single crystal nanostructure can produce chirality by altering the symmetry of the crystal structure.^52,53^ Chirality is a property that distinguishes between right-handed and left-handed objects and arises when a structure has no reflectional symmetry and is not superimposable on its mirror image. This chirality can impact the optical and electronic properties of the crystal, leading to distinct behavior that is dependent on the rotational orientation of the crystal. A variation of such in-plane lattice rotation was previously observed on a micron-sized heterostructure epitaxial nanowire (NW)^52,53^ due to an inhomogeneous strain profile, and in-plane rotation introduced chirality was observed in several other nanostructures.^54,55^ A continuous twist in the in-plane lattice throughout the height of the nanowire was found,^55^ and the observed accumulated twist angle reached up to 21° with a rate of change in continuous twist rotational angle of 0.012° per nanometer height for a typical nanowire with a height of 1.8 μm and a diameter of 64 nm.^55^ For QD1, with a height of 20 nm and a major (length) and minor (width) diameter of 150 and 110 nm, respectively, the lattice rotation varies from 0.6° to 1° with a progressive lattice rotational rate of (0.4°/20 nm) 0.019° per nanometer height, as shown in Figure 4f. For QD2, which has a 144 nm major diameter and 100 nm minor diameter, the lattice rotation varies from 0.3° to 0.6° with 0.015° per nanometer progression in lattice rotation (refer to Figure S13b, SI). To obtain precise quantitative information for QDs, a detailed further investigation of the lattice rotational angle concerning the diameter and height of single QDs is required. However, to the best of our knowledge chiral QDs were not observed before. A QD has significantly better interface quality and shows higher photon–exciton coupling than an NW due to higher-order confinement. Nanoscale chirality can control electrical, optical, and mechanical^35,54^ properties of an NW and produces one of the best nanostructure-based active materials by material engineering in the field of energy harvesting, nanoelectronics, and so on.^13,54,55^ An improved nanoscale-chirality-based single photon^56^ and optoelectronic properties are expected by replacing NWs with QDs as an active source.

## Conclusions

In summary, the simultaneous measurements of XRF and SXDM in transmission X-ray scattering geometry have been applied here to investigate epitaxial QDs. Several QDs can be studied using the presented techniques in quick succession to choose any desired quality in the QD for the development of a single-photon source. SXDM along with complementary optical spectroscopy^2,28^ measurements will help to tune the desired properties of a single QD for improving the pairwise photon indistinguishability in the temporal strings of single photons required for the further advancement of quantum technology.^3,5,9,25^ The obtained results show that both composition and strain profiles have a strong dependence on the in-plane crystallographic directions. They also show that the lattice expansion after a certain composition limit introduces shear strain within the QD. The observed in-plane lattice rotation mediated by the WL during growth enables controlled chiral-QD formation. We believe the directional asymmetry and chirality need to be incorporated into calculations of the optical properties of these QDs for the development of better single-photon sources.

## Methods/Experimental

### Growth of QDs

The InAs-QDs on the GaAs substrate were deposited by MBE techniques using a Veeco Gen III system. A buffer layer of GaAs of 250 nm was grown on the cleaned GaAs (001) wafer at 580 °C followed by the InAs QD layer grown at 515 °C. The growth rates for GaAs and InAs were 1 and 0.027 ML/s. The indium shutter was kept open for 2.8 ML of the InAs layer growth. Different-sized and -shaped self-assembled QDs were formed on the surface during the self-assembly process.

### Microscopic Characterization of the QDs

The epitaxially grown QDs are characterized by AFM and SEM techniques at the DESY NanoLab, as shown in Figure S1b,c. A variation in sizes and shapes is observed in self-assembled QDs. The larger-sized QDs are slightly semiellipsoidal in shape, having major dimensions around 130–150 nm and minor dimensions of 100–110 nm at the base with an eccentricity factor (*e*) of 0.6 to 0.64. The eccentricity factor varies with the height of the QDs. There are a few medium-sized QDs with major dimensions of 75–80 nm and minor dimensions of 70–75 nm with a lower eccentricity of 0.33–0.38. There are highly populated smaller-sized QDs with an equal value of major and minor dimensions of 50 nm with an eccentricity close to zero.

### Characterization of Single QDs

Two larger QDs were selected for our study. The AFM image of the studied QD1 in the article is shown in Figure S1e. It confirms that the major and minor lengths are 151 and 107 nm, respectively. The height of the QD is found to be 20 nm. Different lateral dimensions of the QD are also measured at various heights; for example, the QD has major (minor) lateral dimensions of 83 ± 2 nm (74 ± 2 nm) and 48 ± 2 nm (46 ± 2 nm) at heights of 8 and 16 nm, respectively, from the base. Figure S10a shows the microscopy image of the second QD, QD2; the major and minor base dimensions are observed as 144 and 100 nm, respectively, with a height of ∼20 nm.

### X-ray Microscopy

The sample was mounted on a frame of a SiN*x* membrane (Figure S3, SI), which could be fitted with the standard sample holder in beamline P06 at PETRA-III, DESY. Before mounting, substrate crystallographic orientation was measured at beamline P08, PETRA-III, DESY, using a six-cycle goniometer. By observing the crystallographic orientation of (200), (220), (400), and (440) Bragg peaks of the GaAs substrate, the sample was mounted on the sample holder in such a way that the (400) planes remained vertical and parallel to the X-ray beam during SXDM measurements at the P06 beamline at PETRA-III, DESY. The alignment of the sample was again confirmed by observing (400) and (600) Bragg peaks of the substrate at the P06 beamline (Figure S7, SI). The X-ray energy was finely tuned at the indium Kα-absorption edge, 27940 eV, with the help of a fluorescence detector and indium foil. During measurements, energy was tuned to 28 150 eV to get indium Kα (24 210 eV), gallium Kα (9250 eV), and arsenic Kα (10 540 eV) fluorescence signals simultaneously to the fluorescence maps of the sample. Alignments of the QD, tracking of the precise sample movements, and stabilization of the sample against any unwanted drift were managed using feedback from a three-dimensional interferometric setup at the sample-piezo-stage (see Figure S3, SI). A photograph of the experimental setup is also shown in Figure S3.

The three markers and the preselected QDs are relocated by utilizing a guiding marker-based correlative microscopy approach and an in-line optical microscope at the X-ray beamline in a first, rough alignment step. All the QDs were visible in indium Kα fluorescence intensity mapping by performing mesh scans (Figure S8, SI). It also indicates that any size and shape single QD can be chosen for structural investigation without any markers. This makes the measurement protocol easily implementable to find and investigate any individual QD. The scanning was carried out over an area of 600 nm × 600 nm containing only one QD and its surrounding WL.

### Composition Map

The integrated fluorescence intensity around the Kα fluorescence signal of indium (23700–24500 eV) and gallium (9000–9550 eV) has been captured by an energy-dispersive fluorescence detector. The substrate and WL contributions come into the fluorescence intensity when the X-ray hits a region without a QD. On the other hand, the substrate, WL, and QD contribute to the fluorescence intensity when the X-ray hits a region where a QD is present. Therefore, the difference in the fluorescence intensities between a position containing a QD and a position not containing a QD can be approximated as the intensity contribution of the QD. Details of the composition mapping procedure are discussed in Section II: Interpretation of the captured data is in the SI.

### Lattice Map

The lattice values of any particular Bragg intensity are calculated using standard Bragg’s law from the reciprocal (*H*, *K*) vectors, as shown in Figure 4b–d. An average lattice *a*_XRD_^*i*,*j*^ within a specific part of the single QD is achieved by taking the diffraction intensity (*I*_*m*.*n*_^*i*,*j*^) weighted lattice *a*_*m*,*n*_^*i*,*j*^. Here, (*i*, *j*) represents the index in the 2D mesh scan and (*m*, *n*) represents the pixel number of the area detector. The Bragg intensities lying within the red enclosed area (Figure 2a) were taken only during the average lattice calculation for the QD. XRF calculates compositions within the single QD. The XRF-determined composition-dependent lattice can be calculated by *a*_XRF_(*x*^*i*,*j*^) = 5.653 Å + *x*^*i*,*j*^(6.058 – 5.653) Å. The difference in values between *a*_XRD_(*x*) and *a*_XRF_(*x*) gives the strain of the QD unit cell as calculated previously in the article.

## Data Availability

Extended data are available from the corresponding authors upon reasonable request.

## Supporting Information Available

Section I: detailed measurement protocol; Section II: interpretation of the captured data; Section III: investigation on the second QD, QD2 (PDF)

## References

[ref1] Stangl J.; Holý V.; Bauer G. Structural Properties of Self-Organized Semiconductor Nanostructures. Rev. Mod. Phys. 2004, 76, 725 10.1103/RevModPhys.76.725.

[ref2] Marzin J.-Y.; et al. Photoluminescence of Single InAs Quantum Dots Obtained by Self-Organized Growth on GaAs. Phys. Rev. Lett. 1994, 73, 716 10.1103/PhysRevLett.73.716. 10057519

[ref3] Lodahl P.; Mahmoodian S.; Stobbe S. Interfacing Single Photons and Single Quantum Dots with Photonic Nanostructures. Rev. Mod. Phys. 2015, 87, 347 10.1103/RevModPhys.87.347.

[ref4] Ding X.; He Y.; Duan Z. C.; Gregersen N.; Chen M. C.; Unsleber S.; Maier S.; Schneider C.; Kamp M.; Höfling S.; Lu C.-Y.; Pan J.-W. On-Demand Single Photons with High Extraction Efficiency and Near-Unity Indistinguishability from a Resonantly Driven Quantum Dot in a Micropillar. Phys. Rev. Lett. 2016, 116, 020401 10.1103/PhysRevLett.116.020401. 26824530

[ref5] Uppu R.; Pedersen F. T.; Wang Y.; Olesen C. T.; Papon C.; Zhou X.; Midolo L.; Schloz S.; Wieck A. D.; Ludwig A.; Lodahl P. Scalable Integrated Single-Photon Source. Sci. Adv. 2020, 6, eabc8268 10.1126/sciadv.abc8268. 33298444 PMC7725451

[ref6] Gao W. B.; Fallahi P.; Togan E.; Miguel-Sánchez J.; Imamoglu A. Observation of Entanglement between a Quantum Dot Spin and a Single Photon. Nature 2012, 491, 426–430. 10.1038/nature11573. 23151586

[ref7] Benson O.; Santori C.; Pelton M.; Yamamoto Y. Regulated and Entangled Photons from a Single Quantum Dot. Phys. Rev. Lett. 2000, 84, 2513 10.1103/PhysRevLett.84.2513. 11018923

[ref8] Stevenson R. M.; Young R. J.; Atkinson P.; Cooper K.; Ritchie D. A.; Shields A. J. A Semiconductor Source of Triggered Entangled Photon Pairs. Nature 2006, 439, 179–182. 10.1038/nature04446. 16407947

[ref9] Lu C. Y.; Pan J. W. Quantum-Dot Single-Photon Sources for the Quantum Internet. Nat. Nanotechnol. 2021, 16, 1294–1296. 10.1038/s41565-021-01033-9. 34887534

[ref10] Uppu R.; Midolo L.; Zhou X.; Carolan J.; Lodahl P. Quantum-Dot-Based Deterministic Photon–Emitter Interfaces for Scalable Photonic Quantum Technology. Nat. Nanotechnol. 2021, 16, 1308–1317. 10.1038/s41565-021-00965-6. 34663948

[ref11] Kimble H. J. The Quantum Internet. Nature 2008, 453, 1023–1030. 10.1038/nature07127. 18563153

[ref12] De Riedmatten H.; Marcikic I.; Tittel W.; Zbinden H.; Collins D.; Gisin N. Long Distance Quantum Teleportation in a Quantum Relay Configuration. Phys. Rev. Lett. 2004, 92, 047904 10.1103/PhysRevLett.92.047904. 14995410

[ref13] Mahmoodian S.; Lodahl P.; So̷rensen A. S. Quantum Networks with Chiral-Light–Matter Interaction in Waveguides. Phys. Rev. Lett. 2016, 117, 240501 10.1103/PhysRevLett.117.240501. 28009207

[ref14] Uppu R.; Eriksen H. T.; Thyrrestrup H.; Uğurlu A. D.; Wang Y.; Scholz S.; Wieck A. D.; Ludwig A.; Löbl M. C.; Warburton R. J.; Lodahl P.; Midolo L. On-Chip Deterministic Operation of Quantum Dots in Dual-Mode Waveguides for a Plug-and-Play Single-Photon Source. Nat. Commun. 2020, 11, 3782 10.1038/s41467-020-17603-9. 32728025 PMC7391626

[ref15] Chang W.-H.; Chen W.-Y.; Chang H.-S.; Hsieh T.-P.; Chyi J.-I.; Hsu T.-M. Efficient Single-Photon Sources Based on Low-Density Quantum Dots in Photonic-Crystal Nanocavities. Phys. Rev. Lett. 2006, 96, 117401 10.1103/PhysRevLett.96.117401. 16605868

[ref16] Santori C.; Fattal D.; Vučković J.; Solomon G. S.; Yamamoto Y. Indistinguishable Photons from a Single-Photon Device. Nature 2002, 419, 594–597. 10.1038/nature01086. 12374958

[ref17] Knill E.; Laflamme R.; Milburn G. J. A Scheme for Efficient Quantum Computation with Linear Optics. Nature 2001, 409, 46–52. 10.1038/35051009. 11343107

[ref18] Wehner S.; Elkouss D.; Hanson R. Quantum Internet: A Vision for the Road Ahead. Science 2018, 362, eaam9288 10.1126/science.aam9288. 30337383

[ref19] Northup T. E.; Blatt R. Quantum Information Transfer Using Photons. Nat. Photonics 2014, 8, 356–363. 10.1038/nphoton.2014.53.

[ref20] Liu J.; Su R.; Wei Y.; Yao B.; Silva S. F. C. D.; Yu Y.; Smith J. I.; Srinivasan K.; Rastelli A.; Li J.; Wang X. A Solid-State Source of Strongly Entangled Photon Pairs with High Brightness and Indistinguishability. Nat. Nanotechnol. 2019, 14, 586–593. 10.1038/s41565-019-0435-9. 31011221 PMC10941235

[ref21] Schwartz I.; Cogan D.; Schmidgall E. R.; Don Y.; Gantz L.; Kenneth O.; Lindner N. H.; Gershoni D. Deterministic Generation of a Cluster State of Entangled Photons. Science 2016, 354, 434–437. 10.1126/science.aah4758. 27608669

[ref22] Wang H.; He Y.; Li Y.-H.; Su Z.-E.; Li B.; Huang H.-L.; Ding X.; Chen M.-C.; Liu C.; Qin J.; Li J.-P.; He Y.-M.; Schneider C.; Kamp M.; Peng C.-H.; Höfling S.; Lu C.-Y.; Pan J.-W. High-Efficiency Multiphoton Boson Sampling. Nat. Photonics 2017, 11, 361–365. 10.1038/nphoton.2017.63.

[ref23] Santori C.; Fattal D.; Vučković J.; Solomon G. S.; Yamamoto Y. Indistinguishable Photons from a Single-Photon Device. Nature 2002, 419, 594–597. 10.1038/nature01086. 12374958

[ref24] Kuhlmann A. V.; Houel J.; Ludwig A.; Greuter L.; Reuter D.; Wieck A. D.; Poggio M.; Warburton R. J. Charge Noise and Spin Noise in a Semiconductor Quantum Device. Nat. Phys. 2013, 9, 570–575. 10.1038/nphys2688.

[ref25] Lee C. M.; Buyukkaya M. A.; Harper S.; Aghaeimeibodi S.; Richardson C. J.; Waks E. Bright Telecom-Wavelength Single Photons Based on a Tapered Nanobeam. Nano Lett. 2021, 21, 323–329. 10.1021/acs.nanolett.0c03680. 33338376

[ref26] Walther T.; Cullis A. G.; Norris D. J.; Hopkinson M. Nature of the Stranski-Krastanow Transition during Epitaxy of InGaAs on GaAs. Phys. Rev. Lett. 2001, 86, 2381 10.1103/PhysRevLett.86.2381. 11289934

[ref27] Somaschi N.; Giesz V.; De Santis L.; Loredo J. C.; Almeida M. P.; Hornecker G.; Portalupi S. L.; Grange T.; Antón C.; Demory J.; Gómez C.; Sagnes I.; Lanzillotti-Kimura N. D.; Lemaítre A.; Auffeves A.; White A. G.; Lanco L.; Senellart P. Near-Optimal Single-Photon Sources in the Solid State. Nat. Photonics 2016, 10, 340–345. 10.1038/nphoton.2016.23.

[ref28] Benyoucef M.; Zuerbig V.; Reithmaier J. P.; Kroh T.; Schell A. W.; Aichele T.; Benson O. Single-Photon Emission from Single InGaAs/GaAs Quantum Dots Grown by Droplet Epitaxy at High Substrate Temperature. Nanoscale Res. Lett. 2012, 7, 1–5. 10.1186/1556-276X-7-493. 22937992 PMC3494552

[ref29] Dey A. B.; Sanyal M. K.; Farrer I.; Perumal K.; Ritchie D. A.; Li Q.; Wu J.; Dravid V. Correlating Photoluminescence and Structural Properties of Uncapped and GaAs-Capped Epitaxial InGaAs Quantum Dots. Sci. Rep. 2018, 8, 7514 10.1038/s41598-018-25841-7. 29760396 PMC5951952

[ref30] Dey A. B.; Sanyal M. K.; Keane D. T.; Campbell G. P.; Liu B. H.; Farrer I.; Ritchie D. A.; Bedzyk M. J. X-Ray Atomic Mapping of Quantum Dots. Phys. Rev. Mater. 2020, 4, 056002 10.1103/PhysRevMaterials.4.056002.

[ref31] Fry P. W.; Itskevich I. E.; Mowbray D. J.; Skolnick M. S.; Finley J. J.; Barker J. A.; O’Reilly E. P.; Wilson L. R.; Larkin I. A.; Maksym P. A.; Hopkinson M.; Al-Khafaji M.; David J. P. R.; Cullis A. G.; Hill G.; Clark J. C. Inverted electron-hole alignment in InAs-GaAs self-assembled quantum dots. Phys. Rev. Lett. 2000, 84, 733 10.1103/PhysRevLett.84.733. 11017359

[ref32] Liu N.; Tersoff J.; Baklenov O.; Holmes A. L. Jr; Shih C. K. Nonuniform composition profile in In_0.5_Ga_0.5_As alloy quantum dots. Phys. Rev. Lett. 2000, 84, 334 10.1103/PhysRevLett.84.334. 11015904

[ref33] Rosenauer A.; Fischer U.; Gerthsen D.; Förster A. Composition evaluation by lattice fringe analysis. Ultramicroscopy 1998, 72, 121–133. 10.1016/S0304-3991(98)00002-3.

[ref34] Blokland J. H.; Bozkurt M.; Ulloa J. M.; Reuter D.; Wieck A. D.; Koenraad P. M.; Christianen P. C. M.; Maan J. C. Ellipsoidal InAs quantum dots observed by cross-sectional scanning tunneling microscopy. Appl. Phys. Lett. 2009, 94, 021907 10.1063/1.3072366.

[ref35] Rosenauer A.; Fischer U.; Gerthsen D.; Förster A. Composition evaluation of In_x_Ga_1-x_As Stranski-Krastanow-island structures by strain state analysis. Appl. Phys. Lett. 1997, 71, 3868–3870. 10.1063/1.120528.

[ref36] Robinson I.; Harder R. Coherent X-ray diffraction imaging of strain at the nanoscale. Nat. Mater. 2009, 8, 291–298. 10.1038/nmat2400. 19308088

[ref37] Diaz A.; Chamard V.; Mocuta C.; Magalhães-Paniago R.; Stangl J.; Carbone D.; Metzger T. H.; Bauer G. Imaging the displacement field within epitaxial nanostructures by coherent diffraction: a feasibility study. New J. Phys. 2010, 12, 035006 10.1088/1367-2630/12/3/035006.

[ref38] Vartanyants I. A.; Robinson I. K.; Onken J. D.; Pfeifer M. A.; Williams G. J.; Pfeiffer F.; Metzger H.; Zhong Z.; Bauer G. Coherent x-ray diffraction from quantum dots. Phys. Rev. B 2005, 71, 245302 10.1103/PhysRevB.71.245302.

[ref39] Schülli T. U.; Stangl J.; Zhong Z.; Lechner R. T.; Sztucki M.; Metzger T. H.; Bauer G. Direct determination of strain and composition profiles in SiGe islands by anomalous x-ray diffraction at high momentum transfer. Phys. Rev. Lett. 2003, 90, 066105 10.1103/PhysRevLett.90.066105. 12633307

[ref40] Grote L.; Seyrich M.; Döhrmann R.; Harouna-Mayer S. Y.; Mancini F.; Kaziukenas E.; Fernandez-Cuesta I.; Zito C. A.; Vasylieva O.; Wittwer F.; Odstrčzil M.; Mogos N.; Landmann M.; Schroer C. G.; Koziej D. Imaging Cu_2_O nanocube hollowing in solution by quantitative in situ X-ray ptychography. Nat. Commun. 2022, 13, 4971 10.1038/s41467-022-32373-2. 36038564 PMC9424245

[ref41] Sow C.; Sarma A.; Schropp A.; Dzhigaev D.; Keller T. F.; Schroer C. G.; Sanyal M. K.; Kulkarni G. U. Unraveling the Spatial Distribution of Catalytic Non-Cubic Au Phases in a Bipyramidal Microcrystallite by X-ray Diffraction Microscopy. ACS Nano 2020, 14, 9456–9465. 10.1021/acsnano.0c02031. 32491827

[ref42] Doolette C. L.; Howard D. L.; Afshar N.; Kewish C. M.; Paterson D. J.; Huang J.; Wagner S.; Santner J.; van Leeuwen A. T.; Hou L.; Bom V. B.; Weng H.; Kopittke P. M.; Lombi E. Tandem Probe Analysis Mode for Synchrotron XFM: Doubling Throughput Capacity. Anal. Chem. 2022, 94, 4584–4593. 10.1021/acs.analchem.1c04255. 35276040 PMC8943523

[ref43] Schropp A.; Döhrmann R.; Botta S.; Brückner D.; Kahnt M.; Lyubomirskiy M.; Scholz C. O. M.; Seyrich M.; Stuckelberger M. E.; Wiljes P.; Wittwer F.; Garrevoet J.; Falkenberg G.; Fam Y.; Sheppard T. L.; Grunwaldt J.-D.; Schroer C. G. PtyNAMi: Ptychographic Nano-Analytical Microscope. J. Appl. Crystallogr. 2020, 53, 957–971. 10.1107/S1600576720008420. 32788903 PMC7401781

[ref44] Schroer C. G.; Kuhlmann M.; Hunger U. T.; Günzler T. F.; Kurapova O.; Feste S.; Frehse F.; Lengeler B.; Drakopoulos M.; Somogyi A. S.; Simionovici A. S.; Snigirev A.; Snigireva I.; Schug C.; Schröder W. H. Nanofocusing Parabolic Refractive X-Ray Lenses. Appl. Phys. Lett. 2003, 82, 1485–1487. 10.1063/1.1556960.

[ref45] Schroer C. G.; Kurapova O.; Patommel J.; Boye P.; Feldkamp J.; Lengeler B.; Riekel C.; Vincze L.; Küchler M. Hard X-Ray Nanoprobe Based on Refractive X-Ray Lenses. AIP Conf. Proc. 2007, 879, 1295–1298.

[ref46] Stierle A.; Keller T. F.; Noei H.; Vonk V.; Roehlsberger R. Desy Nanolab. J. Large-Scale Res. Facil. 2016, 2, A76 10.17815/jlsrf-2-140.

[ref47] Kret S.; Benabbas T.; Delamarre C.; Androussi Y.; Dubon A.; Laval J. Y.; Lefebvre A. High Resolution Electron Microscope Analysis of Lattice Distortions and In Segregation in Highly Strained In_0.35_Ga_0.65_As Coherent Islands Grown on GaAs (001). J. Appl. Phys. 1999, 86, 1988–1993. 10.1063/1.370998.

[ref48] Rosenauer A.; Gerthsen D.; Van Dyck D.; Arzberger M.; Böhm G.; Abstreiter G. Quantification of Segregation and Mass Transport in In_x_Ga_1–x_As/GaAs Stranski-Krastanow Layers. Phys. Rev. B 2001, 64, 245334 10.1103/PhysRevB.64.245334.

[ref49] Kosarev A. N.; Chaldyshev V. V.; Cherkashin N. Experimentally-Verified Modeling of InGaAs Quantum Dots. Nanomaterials 2022, 12, 1967 10.3390/nano12121967. 35745307 PMC9228084

[ref50] Biasiol G.; Heun S. Compositional Mapping of Semiconductor Quantum Dots and Rings. Phys. Rep. 2011, 500, 117–173. 10.1016/j.physrep.2010.12.001.

[ref51] Kegel I.; Metzger T. H.; Lorke A.; Peisl J.; Stangl J.; Bauer G.; Garcia J. M.; Petroff P. M. Nanometer-Scale Resolution of Strain and Interdiffusion in Self-Assembled InAs/GaAs Quantum Dots. Phys. Rev. Lett. 2000, 85, 1694 10.1103/PhysRevLett.85.1694. 10970591

[ref52] Hammarberg S.; Dagytė V.; Chayanun L.; Hill M. O.; Wyke A.; Björling A.; Johansson U.; Kalbfleisch S.; Heurlin M.; Lauhon L. J.; Borgström M. T.; Wallentin J. High-Resolution Strain Mapping of a Single Axially Heterostructured Nanowire Using Scanning X-ray Diffraction. Nano Res. 2020, 13, 2460–2468. 10.1007/s12274-020-2878-6.

[ref53] Wallentin J.; Jacobsson D.; Osterhoff M.; Borgstrom M. T.; Salditt T. Bending and Twisting Lattice Tilt in Strained Core–Shell Nanowires Revealed by Nanofocused X-ray Diffraction. Nano Lett. 2017, 17, 4143–4150. 10.1021/acs.nanolett.7b00918. 28613907

[ref54] Li C. Z.; Wang L. X.; Liu H.; Wang J.; Liao Z. M.; Yu D. P. Giant Negative Magnetoresistance Induced by the Chiral Anomaly in Individual Cd_3_As_2_ Nanowires. Nat. Commun. 2015, 6, 10137 10.1038/ncomms10137. 26673625 PMC4703844

[ref55] Sutter P.; Wimer S.; Sutter E. Chiral Twisted van der Waals Nanowires. Nature 2019, 570, 354–357. 10.1038/s41586-019-1147-x. 31011183

[ref56] Chen D.; He R.; Cai H.; Liu X.; Gao W. Chiral Single-Photon Generators. ACS Nano 2021, 15, 1912–1916. 10.1021/acsnano.0c10420. 33544585

