# Supplementary material for: Culling a Self-Assembled Quantum Dot as a Single-Photon Source Using X-ray Microscopy
Source: ACS Nano. 2023 Jul 31;17(16):16080–8. doi: 10.1021/acsnano.3c04835 (PMC10763734; doi:10.1021/acsnano.3c04835)
Supplement: Supplementary file 1 — nn3c04835_si_001.pdf [file nn3c04835_si_001.pdf]

Supplementary information

Culling a self-assembled quantum dot as a single-photon source using X-ray microscopy

Arka Bikash Dey[1, *], Milan K. Sanyal[2, *], Andreas Schropp[3], Silvio Achilles[3], Thomas F. Keller[3,4], Ian Farrer[5], David A. Ritchie[6], Florian Bertram[1], Christian G. Schroer[3] and Oliver H. Seeck[1]

[1]Deutsches Elektronen-Synchrotron DESY, Notkestr. 85, 22607 Hamburg, Germany

[2]Surface Physics and Material Science Division, Saha Institute of Nuclear Physics, Kolkata, West Bengal 700064, India

[3]Center for X-ray and Nano Science CXNS, Deutsches Elektronen-Synchrotron DESY, Notkestr. 85, Hamburg 22607, Germany

[4]Physics Department, University of Hamburg, Hamburg 20355, Germany

[5]Department of Electronic and Electrical Engineering, University of Sheffield, Mappin Street, Sheffield S1 3JD, United Kingdom

[6]Cavendish Laboratory, University of Cambridge, J. J. Thomson Avenue, Cambridge, CB3 0HE, United Kingdom

Section-I: Detailed Measurement Protocol.

A. Sample Preparation:

A1. Thin sample preparation:

The diffraction data from a single quantum dot (QD) is weaker in intensity as compared to that of the substrate. In transmission geometry, the only way to reduce diffraction intensity of the substrate is to have a thin sample; reduced substrate thickness also improves X-ray transmission. One can either use a commercial thin wafer or reduce the wafer thickness after the formation of QDs on it using techniques like controlled ion milling [1], wet lapping [2], or sample lift-out using a focused-ion beam [3].

In our study, we reduced the wafer thickness from 500 µm to 40 µm (**Figure S1a**) using controlled wet lapping with fine diamond nanoparticles from the backside of the wafer. It ensures that approximately 70% the X-rays can penetrate through the wafer, which can be calculated from the X-ray transmission coefficient of the investigated material (here, GaAs) as shown in **Figure S1b**. A removable glue layer protected the sample surface during lapping. The glue was subsequently removed using an acetone solution, followed by cleaning with isopropanol. The choice of wafer thickness is critical for measuring epitaxially grown samples in the transmission scattering geometry.

A2. Microscopy Examination:

Before and after thinning the sample, one has to use microscopy techniques such as scanning electron microscopy (SEM) or scanning tunneling microscopy (STM) [4] to examine the sample surface.

In our study, we carefully thinning down the wafer while preserving the integrity of the quantum dots (QDs) to ensure accurate characterization in the experimental setup. **Figure S1c** represents the scanning electron microscopy (SEM) examination on the QD sample before the sample preparation process. After the preparation process, we again performed the SEM characterization as shown in **Figure S1d** which revealed that the different-sized and shaped QDs remained intact. Larger-sized QDs exhibited consistent major and minor axis dimensions and a consistent eccentricity ranging from 0.6 to 0.64. Atomic force microscopy (AFM) was also used to investigate the samples. **Figure S1e** confirms that the major and minor lengths are 151 nm and 107 nm, respectively for QD1. The height of the QD1 is found to be 20 nm. Different lateral dimensions of the QD are also measured at various heights; for example, the QD1 has major (minor) lateral dimensions of 83 ± 2 nm (74 ± 2 nm) and 48 ± 2 nm (46 ± 2 nm) at height 8 nm and 16 nm respectively from the base.

A3. Marking Regions of Interest:

If a specific region of interest (ROI) on the sample is desired, suitable markers [5] can be deposited. These markers aid in identifying the ROI during measurements at the synchrotron radiation beamline.

In our study, the ROI of the QD sample was marked with two larger 'L-shaped' markers labeled as 'A' in **Figure S2a**. The lengths of these 'L' markers were 80 microns, 40 microns, and 20 microns, as shown in **Figure S2b**. Additionally, smaller 'L' shaped markers labeled as 'B' in **Figure S2a**, with lengths of 30 microns, 20 microns, and 10 microns, were used.

Note: For self-assembled nanoparticles, marker deposition is not necessary as the nanoparticles are distributed throughout the substrate. However, markers can still be used to select specific regions of interest for measurement.

B. At the Synchrotron Radiation beamline:

B4. Sample Mounting and Alignment:

To achieve accurate measurements in the transmission scattering geometry, a specially designed sample stage was utilized, as described in reference [6].

In our study, the sample was mounted on a ball lens retroreflector (Edmund Optics) with a diameter of 10 mm, as shown in **Figure S3b**. **Figure S3a** illustrates the setup, which utilized a three-dimensional interferometric system at the sample-piezo stage to monitor the sample's position with high accuracy. This setup enabled the reception of interferometer signals through 20 m long optical fibers, which were connected to the interferometer controller (PicoScale, SmartAct GmBH). A comprehensive description of this setup can be found in a previously published work [6]. This setup facilitated the alignment of quantum dots (QDs) and allowed for tracking of the sample movements while stabilizing against drift. The piezo stage enabled precise translational movements necessary for nanoscale measurements. The 3D interferometer feedback controller provided sub-nanometer precision measurements of the sample position, accounting for any deviations from the nominal values induced by the piezo stage. In our study, we adopted the transmission scattering geometry, with the substrate facing the incident X-ray beam and the QDs oriented towards the detector, as depicted in **Figure 1a**. This configuration enabled the characterization of individual QDs and facilitated the analysis of various epitaxial nanostructures. Leveraging the advantages

of this setup, researchers can gain comprehensive and efficient insights into nanoscale objects.

B5. Selection of X-ray Energy:

The choice of X-ray energy is critical for effective simultaneous X-ray fluorescence (XRF) and X-ray diffraction (XRD) measurements. To capture the fluorescence signals from all elements present in the sample, it is essential to select an X-ray energy above the absorption edge of all elemental Kα lines due to strong fluorescence yield. If the X-ray energy falls below the Kα absorption edge of any element in the sample, then Lα absorption edge can be used depending on the fluorescence yield.

In our study, we specifically focused on measuring the fluorescence signal from the element indium that form the QDs. We fine-tuned the X-ray energy above the indium Kα absorption edge at 27940 eV by measuring ion chamber count after inserting an indium foil in the X-ray beam path as shown in **Figure S4a**. During measurements, the energy was adjusted to 28150 eV to simultaneously detect the fluorescence signals of indium Kα (24210 eV), gallium Kα (9250 eV), and arsenic Kα (10540 eV) in the sample. We have mapped indium kα fluorescence signal from the same region of the sample once at X-ray energy of 28150 eV (**Figure S4b**) and another at 27900 eV (**Figure S4c**). At X-ray energy above the Kα fluorescence edge shows the presence of the QDs clearly. The selection of appropriate X-ray energy and accurate measurement of fluorescence signals from relevant elements are crucial in this study. Tuning of X-ray energy serves as an important protocol for similar investigations.

B6. Nano-Focused X-ray Beams:

Nano-focused X-ray beams are indispensable for conducting precise measurements in nanoscale studies [6-8]. The availability of nano-focused beams at various synchrotron beamlines, as well as the size of the investigated nano-object, determine the choice of the actual beam size. Several synchrotron beamlines, including PETRA-III's P06 hard X-ray Nano-probe beamline [6-7], MAX-IV's Nano-max beamline [9], ESRF's ID01 and ID16A Nano-imaging beamlines [10-11], APS's 26-ID-C hard X-ray nanoprobe beamline [12], Spring-8's BL39XU hard X-ray nano spectroscopy station, and few other synchrotron beamlines offer advanced capabilities for nanoscale characterization. These beamlines employ various techniques such as nano-focused lenses, multilayer Laue lenses, and zone plates to achieve precise focusing of X-ray beams to desired sizes. Ongoing advancements in X-ray optics hold the promise of even smaller X-ray beam sizes in the near future, further enhancing the spatial resolution of nanoscale investigations. By utilizing these synchrotron beamlines and state-of-the-art focusing techniques, researchers gain access to nano-focused X-ray beams tailored to their specific experimental requirements. This precise focusing capability opens up new avenues for studying nanomaterials and nanostructures with exceptional resolution and sensitivity.

In our study, a set of two nano focusing X-ray lenses (NFLs) made of silicon was employed to create the nano-focused X-ray beam [7-8]. These lenses were integrated into the nanoprobe instrument at beamline P06 at PETRA III (PtyNAMi) [6]. The lenses were aligned in a crossed geometry, enabling the horizontal and vertical focusing of the X-ray beam. The horizontal lens consisted of N_h = 318 single parabolic refractive lenses, with a radius of curvature of R_h = 6.562 µm and a geometric full aperture of $2R_{0,h}$ = 26.248 µm. Similarly, the vertical lens comprised N_v = 263 single parabolic

refractive lenses, with a radius of curvature of R_v = 10.343 µm and a geometric full aperture of $2R_{0,v}$ = 41.372 µm. The distance between the two lenses was adjusted to ensure that the horizontal and vertical foci coincided along the optical axis in the same plane at a working distance of WD = 13.5 mm. The resulting X-ray beam was characterized using scanning coherent X-ray microscopy (ptychography) with a 2D diffraction detector (Eiger X 4M, Dectris Ltd.) positioned at a distance of L = 3.015 m from the sample. The detector had a pixel size of p = 75 µm. A Siemens-star resolution test chart by NTT-AT (ATN/XRESO-50HC) with 50 nm thinnest lines and spaces was used to scan the sample laterally over an area of 2.5 µm × 2.5 µm, with a step size of approximately 25 nm and an exposure time of 20 ms at each scan point. We found that the resolution is equal to the beam-size in transmission geometry. The complex-valued wave field in the focal plane was retrieved by ptychography, resulting in a beam size of 46 nm (horizontal) × 49 nm (vertical) (FWHM) [see **Figure S5, SI**]. Although slightly larger than the theoretically expected size of 32 nm × 41 nm (FWHM), the beam exhibited similar sizes in both the vertical and horizontal directions. The isotropic beam shape was confirmed by the overlapping vertical and horizontal line profiles displayed in **Figure S5, SI**.

B7. Positioning the Area X-ray Detector:

Positioning an area X-ray detector on a translational and rotational stage, considering Bragg's law and transmission scattering geometry, to accurately capture the diffraction data of interest is an important step.

In our study, for accurately capturing the diffraction data of interest, an area X-ray (XSpectrum) 2M GaAs lambda 2D-detector was used in our study. The detector was placed on a plate mounted on a rotational stage. Initially, the detector was positioned

perpendicular to the X-ray beam, facing the direct X-ray beam, as shown in the schematic in **Figure S6, SI**. The scattering angle (2θ) was determined using Bragg's law, which relates the angle of incidence and the wavelength to the interplanar distance of the crystal lattice. In our case, the (400) Bragg planes of the GaAs substrate were utilized, and the interplanar distance was denoted as d_{400}. To capture the (400) Bragg diffraction, the area detector was moved perpendicular to the X-ray beam path by a distance $s = L\tan(2\theta)$. Subsequently, the detector was rotated by an angle $\omega_{det} = 2\theta$, aligning the diffracted X-rays perpendicularly on the detector surface as shown in **Figure S6, SI**. This positioning strategy allowed us to probe the desired Bragg peaks of the nano-objects under investigation, considering the scattering angles and geometries involved.

B8. Calibration of the Area Detector

Calibration of the area detector needs to be performed with diffraction data from a standard sample.

To calibrate the X-ray area detector, diffraction data from a standard Lanthanum hexaboride (LaB_6) powder sample were measured using the 2M GaAs lambda detector as shown in **Figure S7a**. Given the small size of the X-ray beam, a 2D mesh scan was performed to capture complete power rings from the standard sample. The acquired data were analyzed using the DPDAK software [13], which is freely available from the DESY website. This analysis enabled the calibration of the X-ray area detector, providing the exact angular step per pixel and sample to detector distance. It is important to note that this calibration procedure is a standard practice followed at synchrotron radiation beamlines, and different software options available at various beamlines can be utilized.

B9. In-Line Optical Microscope for Marker Localization

In order to locate the markers, an in-line optical microscope need to be employed, which is commonly available in most nano-focused X-ray synchrotron beamlines.

A guiding marker-based correlative microscopy approach, combined with the in-line optical microscope, was utilized to relocate the three markers and the pre-selected quantum dots (QDs). This initial rough alignment step helped identify the region of interest, minimizing unnecessary large area scans on the samples. Once the region of interest was identified, the optical microscope could be retracted from the X-ray beam path.

B10. Sample Alignment Using Substrate Bragg Peaks

Alignment of the sample by identifying substrate Bragg peaks need to be performed. Substrate Bragg peak signal on X-ray area detector can serve as a reference for finding nanodot's Bragg signal [14].

At beamline P06, the sample was precisely aligned by observing the substrate's (400) and (600) Bragg peaks, as depicted in **Figure S7b**. This figure presents a merged diffraction image collected at the two Bragg angles: 8.96° for the (400) Bragg peak and 13.52° for the (600) Bragg peak, corresponding to q-values of 4.45 $Å^{-1}$ and 6.67 $Å^{-1}$, respectively as shown in **Figure S7c**. A tilt stage with a tilt angle of ± 5° was available to correct for sample tilt during measurements at the P06 beamline. In the presence of tilt, the (400) and (600) Bragg peaks would not appear in the same horizontal line; instead, their joining line would create a tilt angle on the detector. **Figure S7b** confirms that the sample was adequately aligned, as the joining line of the Bragg peaks appears completely horizontal on the detector panel. Additionally, the substrate's (400) Bragg peak served as a reference point of known angles on the detector. In this study, the

(400) GaAs substrate Bragg peak was used as the reference point, with a scattering angle of 17.92° in the horizontal direction with respect to the X-ray beam path. The joining line of the (400) and (600) Bragg peaks of the substrate defined the horizontal baseline on the detector, where q_y = 0, as illustrated in Figure 1a.

B11. Optimization of Energy-Dispersive Fluorescence Signal

Positioning an energy-dispersive fluorescence detector on a separate translational and rotational stage to optimize the fluorescence signal [15-16] is necessary.

To optimize the fluorescent signal, an energy-dispersive fluorescence detector was positioned on a separate translational and rotational stage. This setup allowed independent translation and rotation of the detector to achieve the best signal quality. The fluorescence detector collected full spectra, as illustrated in **Figure S8a**. To obtain elemental mapping, specific regions of interest in the full spectrum were selected. In this study, three regions of interest were chosen, centered around the indium Kα (24210 eV), gallium Kα (9250 eV), and arsenic Kα (10540 eV) regions. After performing a 2D mesh scan on the sample, a python-based code was utilized to generate 2D fluorescence maps for indium, gallium, and arsenic. These maps were obtained by integrating the fluorescence signal at each position and then mapped the integrated intensity at each position (x, y) during the mesh scan. **Figure S8c** clearly demonstrates the visibility of all the quantum dots (QDs) in the indium Kα fluorescence intensity mapping, thereby enabling the investigation of individual QDs without the need for markers. This feature makes the measurement protocol highly practical for the identification and investigation of any individual QD. Indium and gallium fluorescence maps during the 2D mesh scan are shown in **Figure S8c** and **S8d** respectively.

B12. Simultaneous Acquisition of X-ray Nano-Diffraction and X-ray Fluorescence Spectra

During the mesh scan, it is essential to acquire both X-ray nano-diffraction [17] and X-ray fluorescence spectrum [18] at each position. This can be achieved by utilizing standard interface synchronization tools readily available in synchrotron beamlines. To capture the QD signal, the rotational stage needs to scan within a few degrees around the substrate Bragg angle. The lattice of InAs and GaAs represents the extreme values of the Bragg incidence angle in this study. Once the QD signals are located, the synchronization interface between sample scanning and data acquisition ensures the simultaneous acquisition of X-ray diffraction patterns and fluorescence data. Diffraction patterns are acquired at each position of the scan using the area detector, providing valuable information about the lattice structures and orientations of the nanostructures. Simultaneously, X-ray fluorescence signals emitted by the sample are collected at each position using the fluorescence detector. These fluorescence signals provide insights into the elemental composition and distribution within the scanned area. By synchronizing the acquisition of diffraction and fluorescence data, a comprehensive understanding of the structural and elemental characteristics of the nanostructures has been achieved.

In **Figure S9**, we present the reciprocal space mapping of the (400) diffraction intensity when the X-ray hits the portions within the QD (region A in **Figure S9a**), and another summed-up image when the X-ray does not hit the QD (region B in **Figure S9a**). The summed-up diffraction intensity has been shown in **Figure S9b** and **Figure S9c** when the X-ray hits the QDs (enclosed area in the region ‘A’ in **Figure S9a**) and does not hit the QDs (enclosed area in region ‘B’ in **Figure S9a**). Apparently, it is clear that the diffracted signal (white box in **Figure S9b**) coming out from the single QD is quite

higher than the noise level in that same H-K reciprocal space (white box in **Figure S9c**) when the X-ray is not hitting any portions of the QD. To have some quantitative idea about the signal-to-noise ratio, we took the ratio of the integrated intensity within the white box between **Figure S9b** and **Figure S9c**. The ratio is coming out to be around ~ 9.2. This can be considered an optimum good-quality diffraction signal. We have also calculated the standard deviation of the noise in each pixel (from the white enclosed area in **Figure S9c**), which is coming out to be 2 (negligible noise fluctuation).

Section-II: Interpretation of the captured Data.

A. Reciprocal space mapping and Scanning X-ray diffraction microscopy:

Following the calibration of the X-ray area detector using data from the standard LaB6 sample and the substrate's (400) and (600) Bragg peaks, the reciprocal space mapping (RSM) can be constructed with respect to substrate miller indices H and K using Laue conditions. Here, H and K represents the miller indices, basically the reciprocal lattice unit along [100] and [010] directions respectively. In this mapping, the diffraction intensities are displayed in reciprocal space, providing valuable insights into the structural characteristics of the sample. An example of a typical reciprocal space map is depicted in Figure 2a of the main manuscript.

To analyze specific regions of interest within the reciprocal space, one can select a particular area, such as the red box shown in **Figure 2a**, and integrate the diffracted intensity within that region. This integration is performed by summing the intensity values I_{mn} from each pixel (m, n) with that particular region on the area detector.

Mathematically, the integrated intensity can be expressed as $S = \sum_{mn} I_{mn}$, where I_{mn} represents the intensity at pixel (m, n) on the detector.

During the mesh scan conducted on the region containing a single quantum dot (QD), the mesh scan itself can be represented by two variables (i, j), which correspond to two perpendicular directions in the 2D scan. These variables represent the real-space coordinates within the investigated nano-object. At each position (i, j) during the mesh scan, an integrated intensity S is obtained, resulting in a functional dependence of S on (i, j), i.e., $S = S^{i,j}$. By mapping $S^{i,j}$ back to the real-space coordinates (i, j), a comprehensive integrated diffraction map throughout the investigated nano-object is generated. This technique is referred to as scanning X-ray diffraction microscopy (SXDM). The results of SXDM performed on the red box region (depicted in **Figure 2a**) within the reciprocal space map are presented in **Figure 2b**.

By employing reciprocal space mapping and SXDM, detailed information regarding the structural characteristics and spatial distribution of the nanostructures can be extracted, contributing to a comprehensive understanding of the sample's properties.

B. Composition map from elemental XRF data:

The integrated fluorescence intensity around the Kα fluorescence signal of indium, ($23700 - 24500$ eV), and gallium, ($9000 - 9550$ eV was obtained using an energy-dispersive fluorescence detector. **Figure S8b** presents a schematic cross-sectional view of the quantum dot (QD), illustrating the X-ray path through the substrate (GaAs) and the QD. At position-1, the X-ray passes through the substrate and the wetting layer (WL), contributing to the fluorescence intensity in the absence of the QD. Conversely, at position-2, the X-ray traverses the substrate, WL, and QD, leading to the fluorescence intensity contribution from the substrate, WL, and QD region. The

difference in fluorescent intensities between position-2 and position-1 corresponds solely to the QD contribution. To efficiently calculate the gallium ($Y_{Ga}^{i,j}$) and indium ($Y_{In}^{i,j}$) contributions from the QD at position 2, the following expression can be used,

$$Y_{In}^{i,j} = Y_{In}^{i,j} - Y_{In}^{i1,j1}$$

$$Y_{Ga}^{i,j} = Y_{Ga}^{i,j} - Y_{Ga}^{i1,j1}$$

Here, the position-1 is denoted by superscript $(i1, j1)$ and position-2 is denoted by superscript (i, j). Figure S3c demonstrates that the fluorescence intensity distribution of gallium remains relatively unchanged during the mesh scan around the single QD, indicating its uniform presence throughout the substrate and its significant contribution from the substrate itself.

The fluorescent intensity[2] depends on Kα fluorescence yield (σ), Indium composition (x), beam size (A) and length (l) of the QD through which X-ray passes through.

$$Y_{In}^{i,j} \sim \sigma_{In}\, x^{i,j}\, A\, l\, f_{E_{In}}$$

$$Y_{Ga}^{k} \sim \sigma_{Ga}\, (1 - x^{i,j})\, A\, l\, f_{E_{Ga}}$$

Here, $\sigma_{In} = 0.853; f_{E_{In}}$ = 0.99979; $\sigma_{Ga} = 0.507; f_{E_{In}}$ = 0.99892.

By taking ration between the fluorescent intensities,

$$\frac{Y_{In}^{i,j}}{Y_{Ga}^{i.j}} = \frac{\sigma_{In}\, x^{i,j}\, A\, l\, f_{E_{In}}}{\sigma_{Ga}\, (1 - x^{i,j})\, A\, l\, f_{E_{Ga}}}$$

$$= \frac{\sigma_{In}\;\; x^{i,j}\; f_{E_{In}}}{\sigma_{Ga}\, (1 - \; x^{i,j})\, f_{E_{Ga}}}$$

Therefore, Indium composition could be calculated as,

$$x^{i,j} = \frac{Y_{In}^{i,j}\ \sigma_{Ga} f_{E_{Ga}}}{\left[Y_{Ga}^{i,j}\ \sigma_{In}\ f_{E_{In}} + Y_{In}^{i,j}\ \sigma_{Ga}\ f_{E_{Ga}}\right]}$$

This equation provides a means to determine the indium composition within the QD based on the measured fluorescent intensities and the known fluorescence properties of indium and gallium.

C. Lattice map from RSM and XRF

The lattice values corresponding to specific Bragg intensities are determined using Bragg's law based on reciprocal (H, K) vectors, as illustrated in **Figure 4(b-d)**. To obtain an average lattice value ($a_{XRD}^{i,j}$) within a particular region of the single quantum dot (QD), the diffraction intensity ($I_{m,n}^{i,j}$) is weighted by the lattice value $a_{m,n}^{i,j}$. This can be expressed as:

$$a_{XRD}^{i,j} = \sum_{m,n} I_{m,n}^{i,j}\ a_{m,n}^{i,j}$$

In contrast, X-ray fluorescence (XRF) analysis provides information about the composition of the single QD. The composition-dependent lattice parameter determined by XRF, $a_{XRF}\,(\,x^{i,j}\,)$, can be calculated using Vegard's law as follows: $a_{XRF}\,(\,x^{i,j}\,)\ =\ 5.653\ \text{Å} +\ x^{i,j}\ (6.058 - 5.653)\ \text{Å}$. The difference between $a_{XRD}^{i,j}$ and $a_{XRF}\,(\,x^{i,j}\,)$ provides the strain of the QD at each position (i, j) during the 2D mesh scan, as depicted in Figure 3b in the main manuscript.

Section-III: Investigation on the second QD, QD2.

Another larger single QD was also randomly chosen and studied similarly to the first single QD studied in the main manuscript. The investigated results for the second QD are shown here in the supplementary information to ensure that the results are systematic and consistent for any randomly chosen larger-sized single QD. **Figure S10a** shows the microscopy image of the second QD; the major and minor base dimensions are observed as 144 nm and 100 nm, respectively, with a height of ~ 20 nm. The second QD (refer to the black ellipse in **Figure S10b**) and the surrounding interface are also found by the indium Kα fluorescence intensity mapping with a 50 nm X-ray beam. The QD2 and its interface with the substrate can be seen as red (higher intensity) and yellow (medium intensity), respectively, in the XRF intensity map. **Figure S10c** shows the reciprocal space mapping of (400) Bragg diffraction peak data from the QD2. The substrate gives an intense Bragg peak (the blue box in **Figure S10c**), and the QD2 also gives relatively less intense scattered peaks at lower -values (the red box in **Figure S10c**). The QD and the surrounding interface are visible in SXDM measured over these less intense peaks, as shown in **Figure S10d**. The in-plane-area observed (red center, **Figure S10d**) by SXDM is found to be slightly larger than the area that contains higher indium (red center, **Figure S10b**) and the physical boundary of the second QD (refer to **Figure S10a**). This observation is similar to what we observed for the first QD.

We also observe directional anisotropies in the in-plane distribution of composition map (**Figure 11e**) and strain map (**Figure S11f**) within the QD2. The QD2 also shows that the indium loading within QD is different along crystallographic directions; this is a similar result that we observed for the first QD. **Figures S11c and S11d** shows the composition distribution in different crystallographic directions and **Figures S11e and**

S11f shows the strain distribution in different crystallographic directions. It is clear from the composition and strain mapping that the composition and strain profiles in the crystallographic directions along [110] and [010] are pretty different (gaussian-like profile) from that observed in direction along [100] (flat-profile) within the QD2. The nature of the indium concentration line profiles remains very different in various in-plane directions even after integrating over the entire QD (refer to **Figures S11g** and **S11h**) - this lateral anisotropy is also similar like what we have observed for the first QD.

The corresponding (400) diffraction signals of the three different portions, central (C), left-upper (LU), right-lower (RL), (**Figure S12a**) of QD are shown in the reciprocal space (H-K) map in **Figure S12b-d**. It also shows a similar result to the first QD. The 'C' region exhibits mainly a larger lattice (5.82 Å and 5.83 Å) (**Figure S12b**), and the peripheral regions, 'LU' and 'RL', show primarily lattice constants of 5.80 Å and 5.81 Å (**Figure S12c-d**) due to a relatively lower indium content, though the traces of 5.82 Å are also obtained in both the QD positions. Similar to the first QD, the second QD also exhibits (400) diffraction signals at nonzero K-values (**Figure S12b-d**), and the K-values approach zero as indium concentration decreases to reach the GaAs substrate (400) peak as shown in **Figure S13a**. The second QD also shows a progressive rotation of the in-plane lattice (Figure **S13a-b**) and introduces a chirality within the QD2. The (400) diffraction signals from the QD2 follows the grey arrow which is not parallel to the purple arrow, indicating continuous twist throughout the height of the QD2. The progressive in-plane rotation is demonstrated schematically in **Figure S13a**, and the corresponding diffraction signals (blue color) are observed in the H-K space, as shown in **Figure S13b**. The light-blue-colored arrow indicates the linear trend, which intersects the K=0 line at H = 3.94, which corresponds to the indium composition of

$x_0 = 0.21 \pm 0.01$. The observed value is the same as that observed for the first QD. For QD2, the lattice rotation varies from 0.3° to 0.6° with 0.015° per nanometer progression in lattice rotation (refer **Figure S13b**). The QD-2 also shows chirality in the crystal orientation.

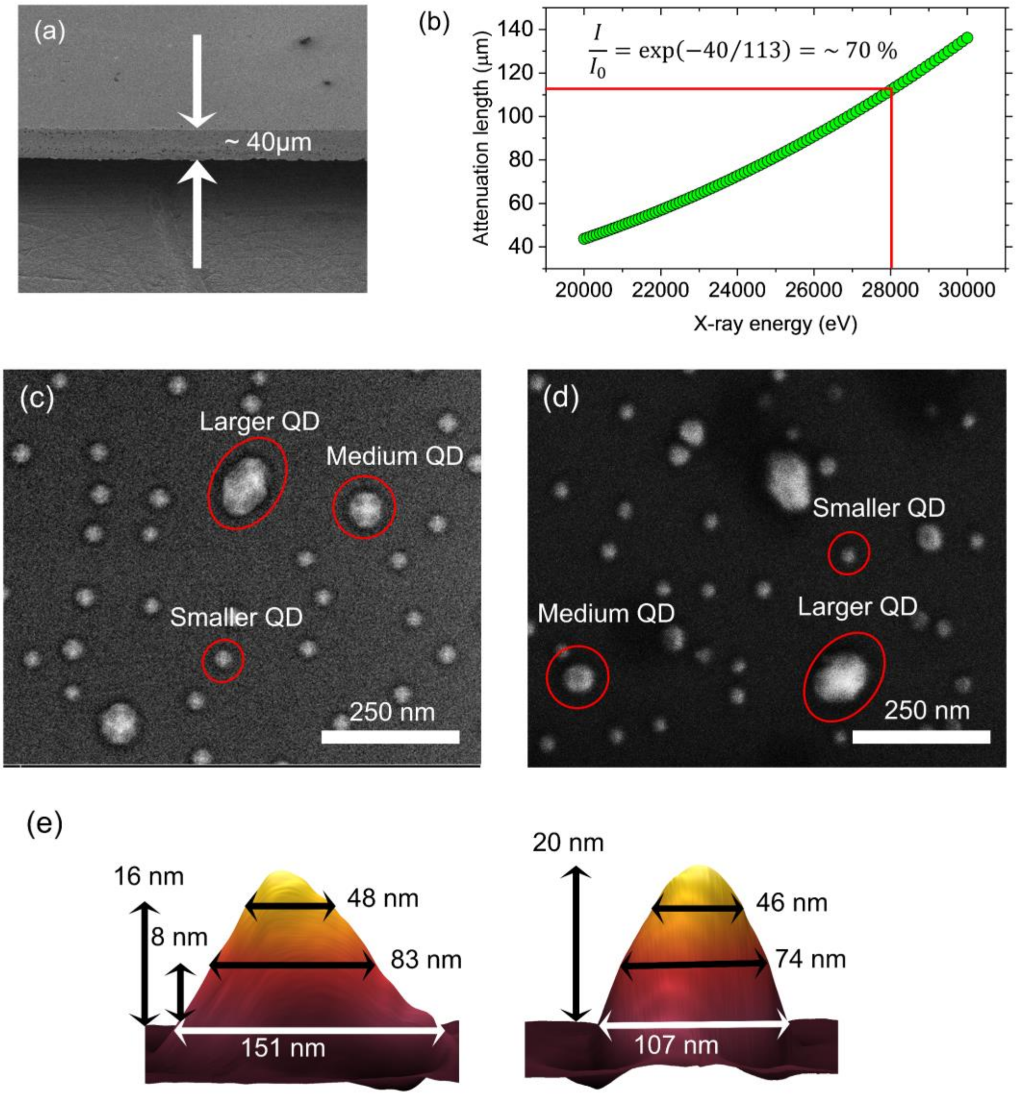

Figure S1. a, The thickness of the substrate was reduced to approximately 40 µm. **b,** X-ray attenuation length through GaAs at various X-ray energies, demonstrating that approximately 70% of X-rays can transmit through the 40 µm thick GaAs substrate. **c,** Scanning electron microscope (SEM) image of the self-assembled QDs after growth. **d,** SEM image of the self-assembled QDs after sample preparation. **e,** Atomic force microscope (AFM) image showing the maximum height of 20 nm for the selected QD1, with a 3D side view depicting the elongated and perpendicular directions.

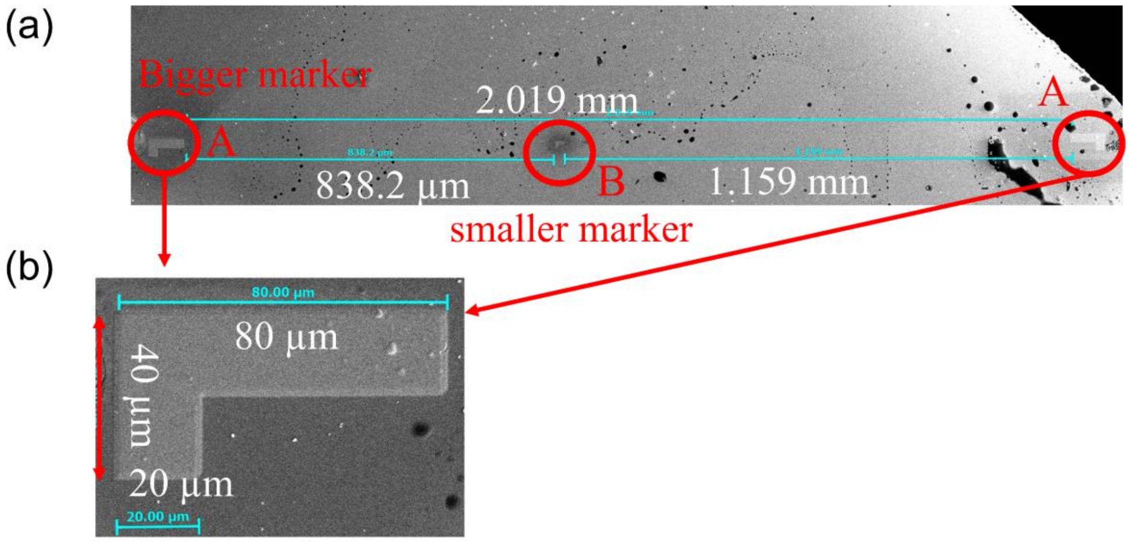

Figure S2. a, The region of interest (ROI) was marked with two ‘L-shaped’ markers as indicated by ‘A’. **b,** The lengths of the bigger ‘L’ marker are 80 µm, 40 µm and 20 µm. The small shaped marker ‘L’ has lengths of 30 µm, 20 µm, and 10 µm as indicated as ‘B’ in Figure S2a.

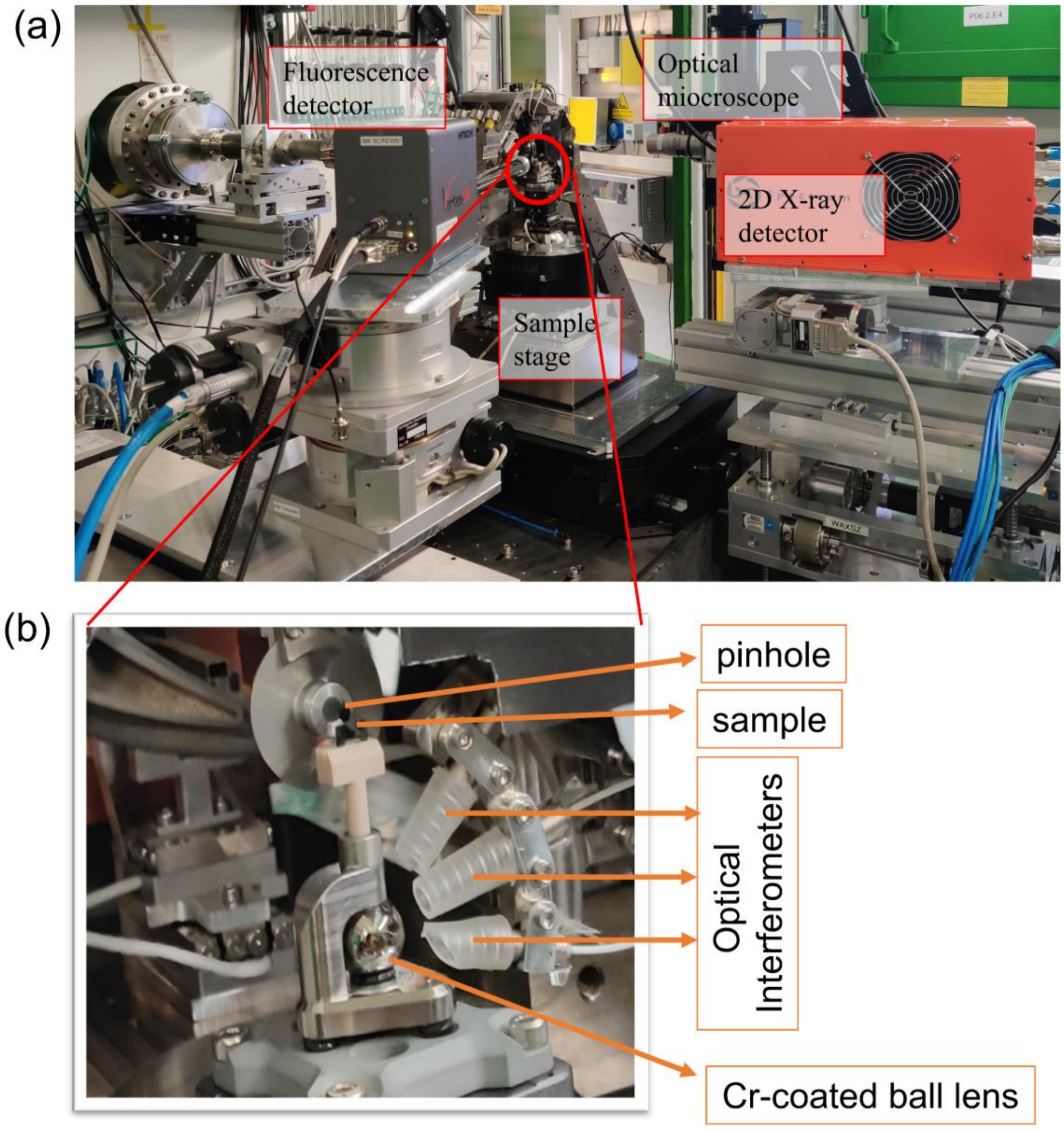

Figure S3. a, Experimental set-up at the P06 beamline, Petra-III, DESY. Elemental fluorescence and scattered photons are simultaneously detected by the fluorescence and area X-ray (XSpectrum) 2M GaAs lambda 2D-detector. **b,** The sample is mounted on a ball lens retroreflector (Edmund Optics) for precise feedback. A three-dimensional interferometric system is utilized through 20 m long optical fibers, which are connected to the interferometer controller (PicoScale, SmartAct GmBH) [6].

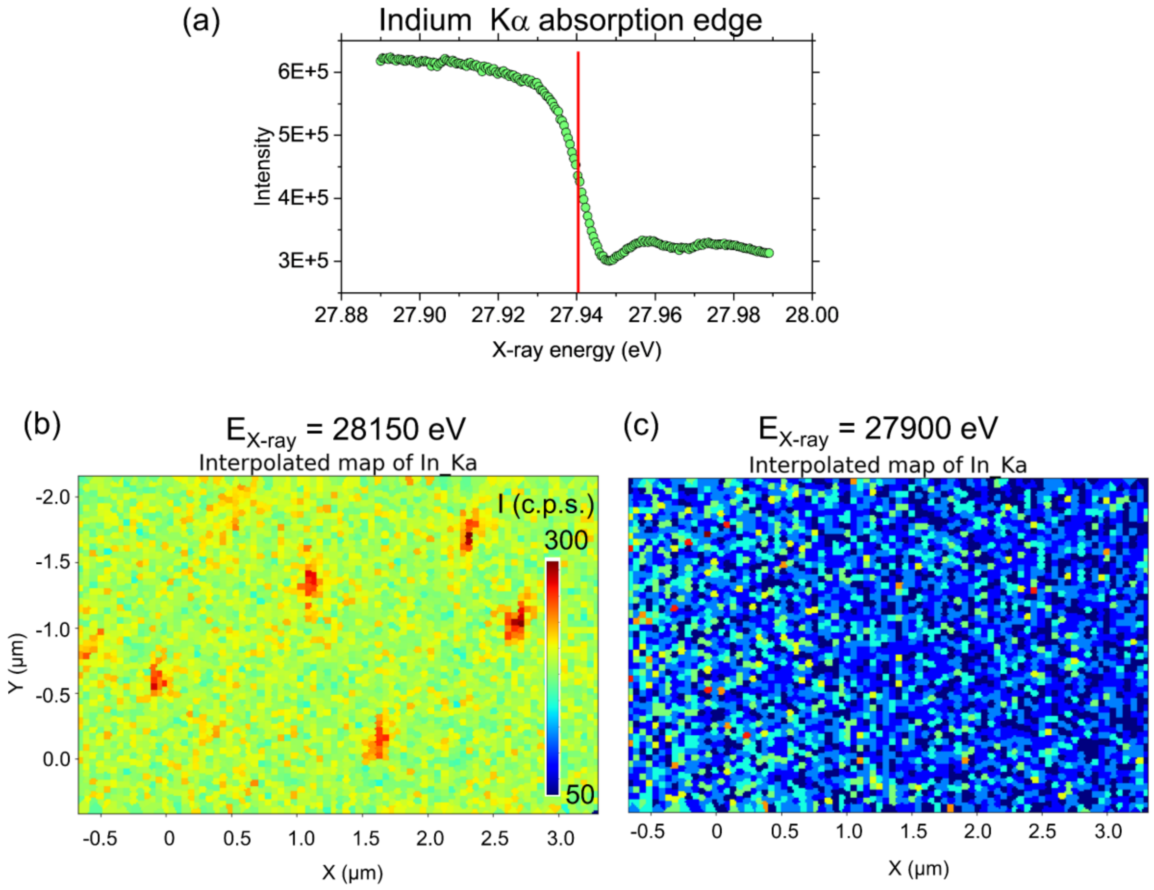

Figure S4. a, Measurement of ion chamber intensity after inserting an indium foil in the X-ray beam path to measure the indium Kα absorption edge. A sharp decrease in X-ray intensity is observed at the indium Kα absorption edge during the X-ray energy scan. **b,** Indium Kα fluorescence mapping obtained when the X-ray energy is set at 28150 eV (~200 eV above the indium Kα absorption edge). **c,** Indium Kα fluorescence mapping obtained when the X-ray energy is set below the indium Ka absorption edge at 27900 eV.

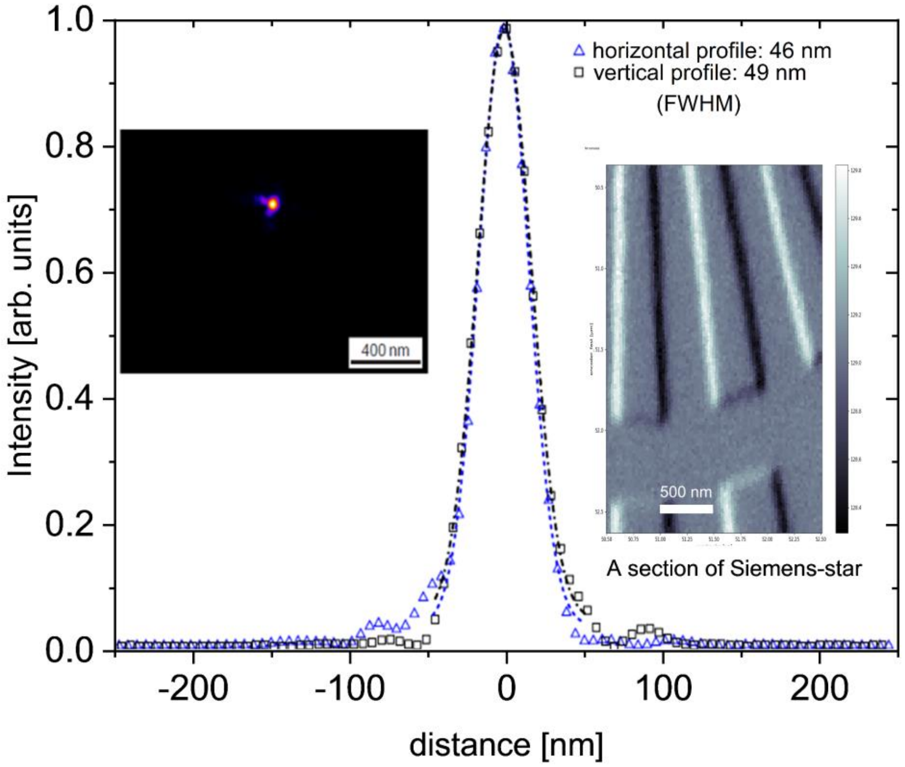

Figure S5. X-ray beam intensity in the focal plane as determined by ptychography (inset). Line profiles were extracted in both the vertical and horizontal directions of the beam. The right inset illustrates the use of a standard Siemens-star (ATN/XRESO-50HC) with 50 nm thinnest lines and spaces for measuring the spatial resolution using scanning coherent X-ray microscopy (ptychography) with a 2D diffraction detector (Eiger X 4M, Dectris Ltd.). Further details on the measurement of spatial resolution using the standard Siemens-star are provided in [6].

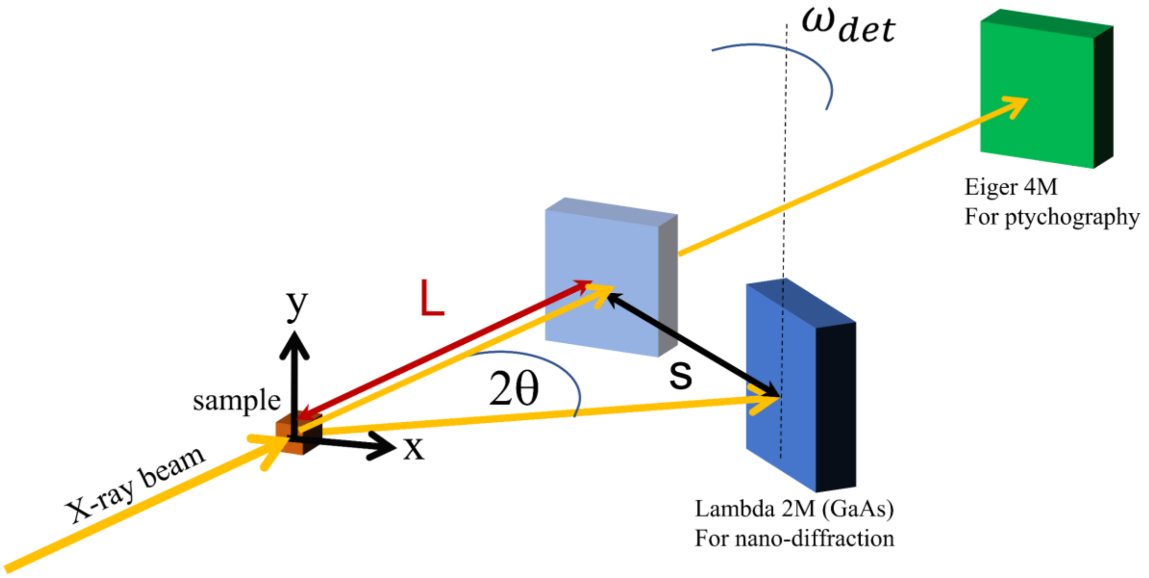

Figure S6. Schematic representation of the 2D detectors used in the experimental setup. The position of the X-ray (XSpectrum) 2M GaAs lambda 2D-detector for diffraction measurement is indicated, as well as the position of the 2D diffraction detector (Eiger X 4M, Dectris Ltd.) for scanning coherent X-ray microscopy (ptychography) measurement.

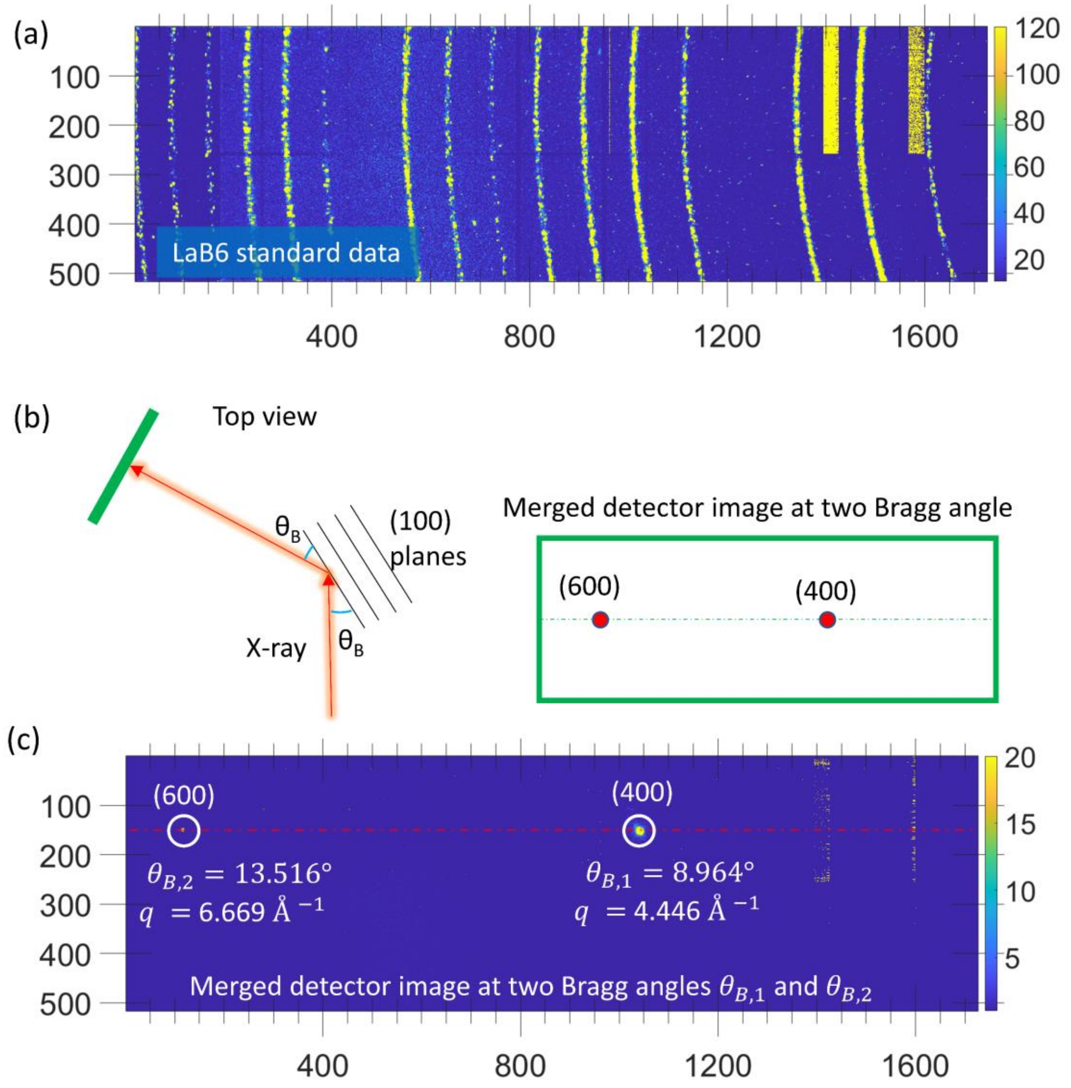

Figure S7. a, Powder diffraction pattern of a standard LaB6 sample used for calibration of the 2D area detector. **b,** Schematic illustration showing the higher-order (100) planes satisfying the Bragg conditions for the (400) and (600) Bragg peaks. **c,** The top channel of the detector captures the merged diffraction signals at two Bragg conditions, (400) and (600), occurring at Bragg angles $\theta_{B,1} = 8.964°$ and $\theta_{B,2} = 13.516°$ respectively. The red-dotted line represents the [100] direction of the GaAs substrate.

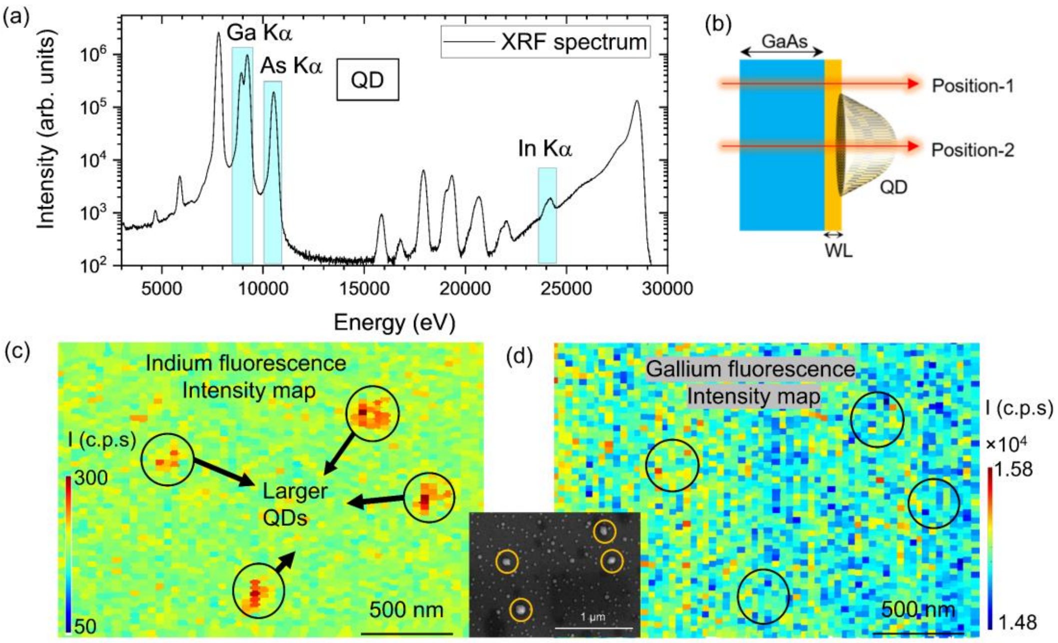

Figure S8. a, Full X-ray fluorescence (XRF) spectrum acquired at a position where the X-ray beam interacts with the quantum dot (QD). **b,** Schematic representation of the QD structure, illustrating the path of the X-ray as it passes through the substrate (GaAs) and interacts with the QD. At position-1, the X-ray passes through the substrate and the window layer (WL), while at position-2, it passes through the substrate, WL, and QD. **c,** Mapping of the Indium Kα fluorescence intensity, revealing a pre-selected region (inset) identified by scanning electron microscopy (SEM). The inset shows the selected region containing four larger-sized self-assembled QDs, each exhibiting slightly different size and shape. **d,** Mapping of the Gallium Kα fluorescence intensity obtained during the same mesh scan.

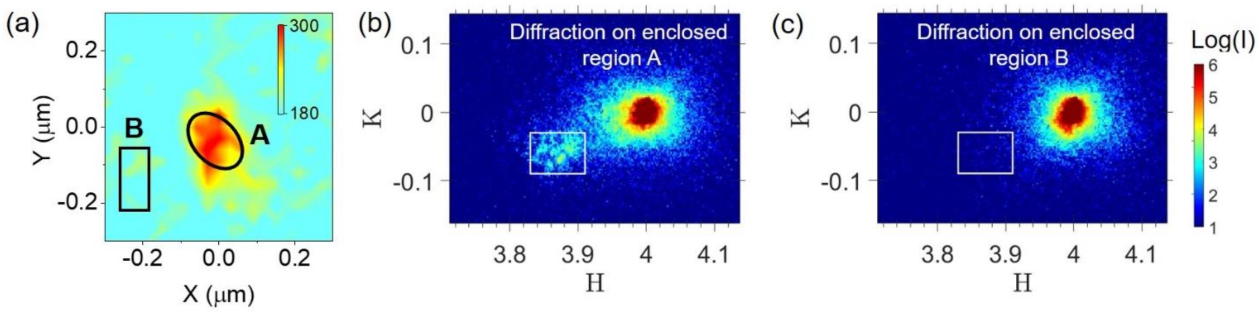

Figure S9. **a,** Indium XRF imaging during the scan; the region 'A' indicates the region when the X-ray hits the QD1 and the region 'B' indicates a region when the X-ray does not hit the QD1. **b,** Sumed-up diffraction when X-ray hits the region 'A'. **c,** Summed-up diffraction when X-ray hits the region 'B'. Both figures b and c have the same color bar.

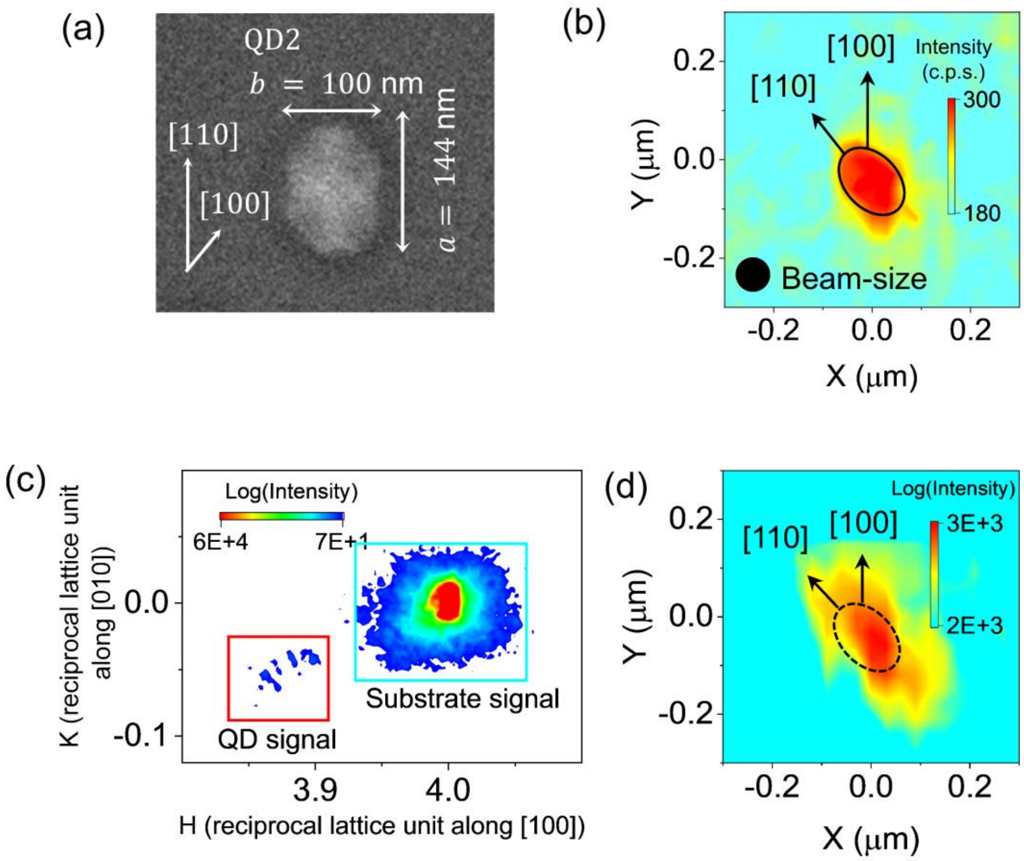

Figure S10. a, SEM image of the second QD (QD2) shows lateral dimensions of 144 nm × 100 nm. **b,** Indium Kα fluorescence intensity mapping of the QD2 during mesh scan around the QD2. The black ellipse represents the position of the QD2. **c,** Summed diffraction signals when the x-ray beam hits the regions within the QD2, within the black ellipse in (b). **d,** Scanning X-ray diffraction microscopy of the Q-space enclosed within the red box in (c).

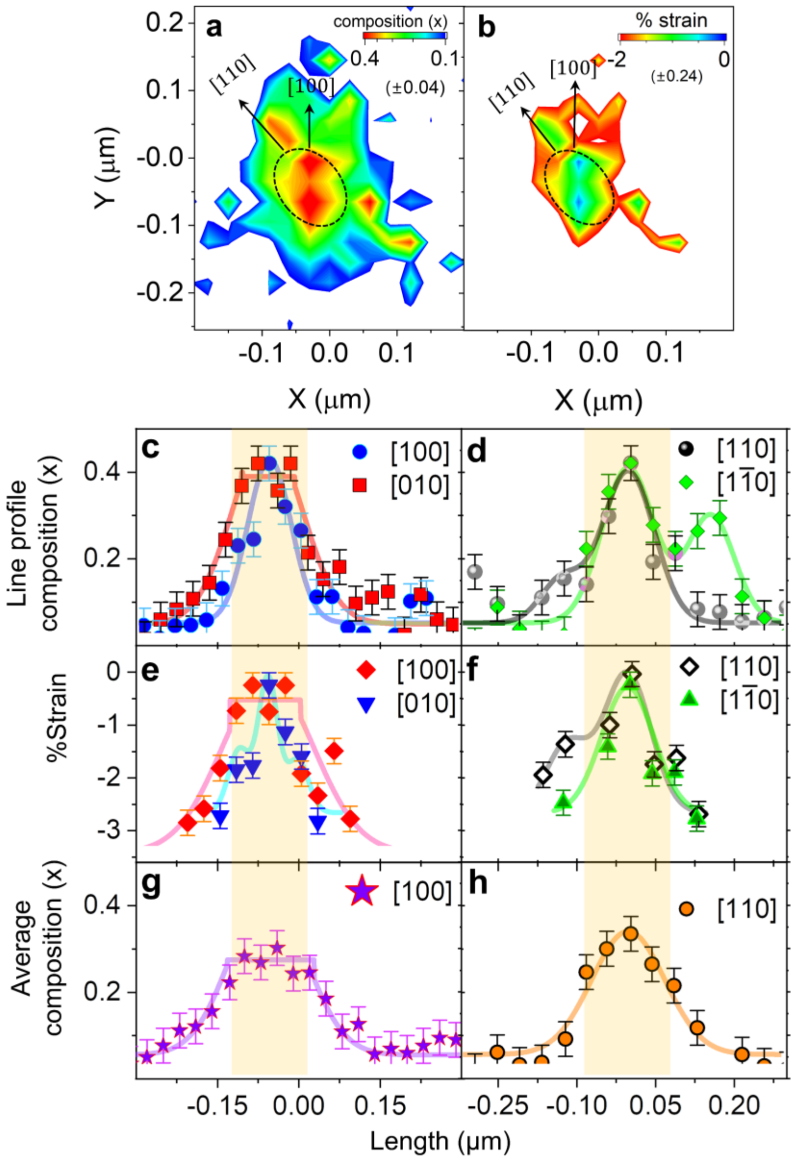

Figure S11. a, Indium composition mapping within the QD2 and its interface as obtained from elemental (Indium and Gallium) X-ray fluorescent signals for the QD2. **b,** It shows absolute in-plane strain (in %) mapping within the second QD2 and its interface. **c,** Line (~ 30 nm width) profiles of the Indium composition along **c,** [100] and [010], **d,** [110] and [1$\bar{1}$0], passing through the center of the QD2. The average Indium compositions of the QD2 and its interface along **e,** [100] and **f,** [110] directions. The yellow highlighted regions represent the regions inside the QD2. Line (~ 50 nm width) profiles of average composition within the QD2 and its interface are plotted along **g,** [100] **h,** [110] directions.

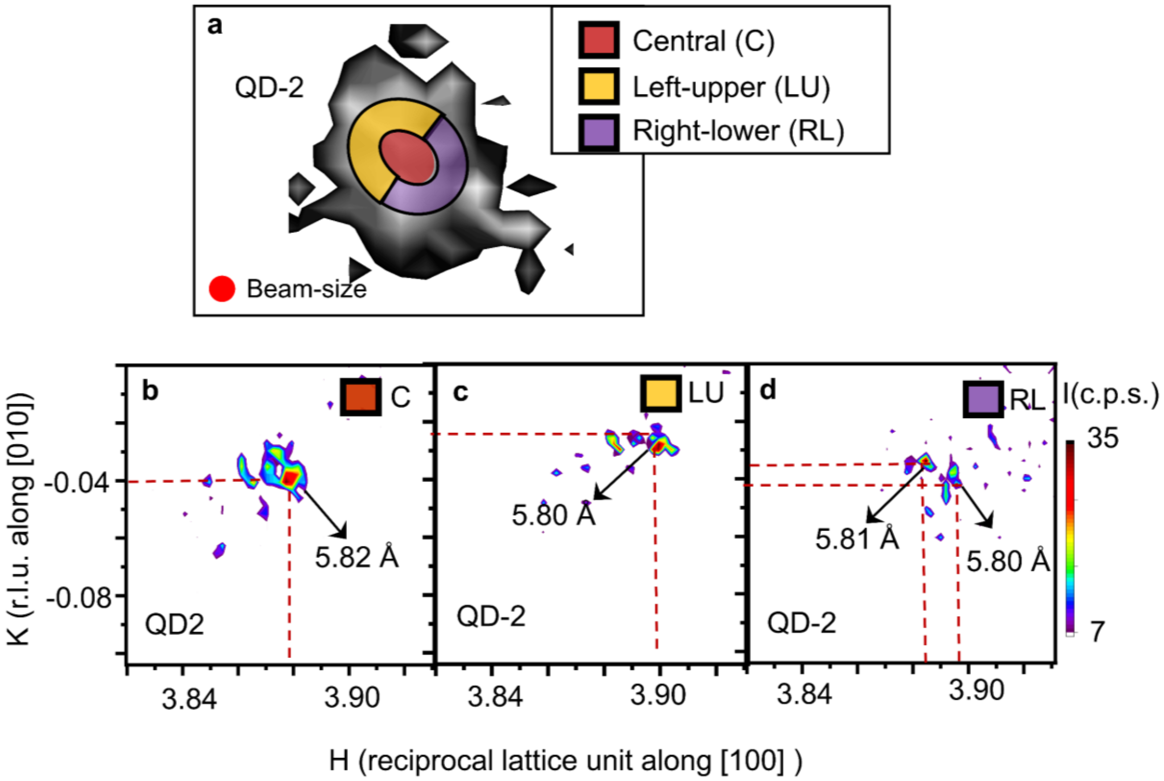

Figure S12. a, Three different portions (three colors) of the QD (QD2) are shown to demonstrate the (400) diffraction intensities from the different portions. The inset shows the atomic force microscopy (AFM) image to demonstrating how X-ray hits different portions in a cross-sectional view. **b,** central (C), **c,** the left-upper (LU), and **d,** the right-lower (RL) portions of the QD2 using the (400) diffraction.

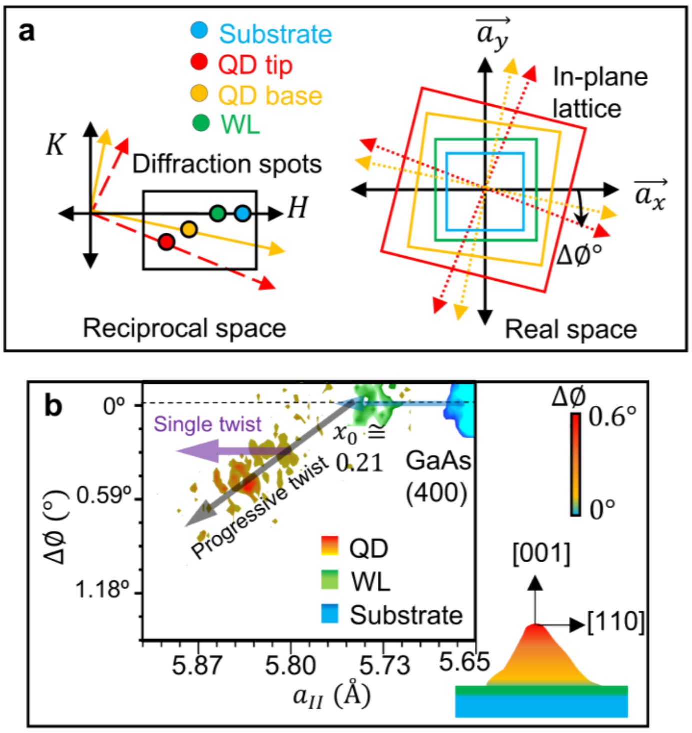

Figure S13. a, A schematic represents how the diffraction spots are related to the in-plane lattice of the QD2, WL and substrate. **b,** Lattice expansions and angular rotation of the QD2 (red-yellow), wet-layer (green), and substrate (blue) varies linearly. The schematic shows the vertical positions of the in-plane lattices within the QD2 (in a cross-sectional view).

References

1. Chang, Y.; Lu, W.; Guénolé, J. et al. Ti and Its Alloys as Examples of Cryogenic Focused Ion Beam Milling of Environmentally-Sensitive Materials. Nat. Commun. 2019, 10, 942.

2. Pinel, S. et al. Mechanical Lapping, Handling, and Transfer of Ultra-Thin Wafers. J. Micromech. Microeng. 1998, 8(4), 338.

3. Munroe, P. R. et al. The Application of Focused Ion Beam Microscopy in the Material Sciences. Mater. Charact. 2009, 60(1), 2-13.

4. Kumah, D.; Shusterman, S.; Paltiel, Y. et al. Atomic-Scale Mapping of Quantum Dots Formed by Droplet Epitaxy. Nat. Nanotech. 2009, 4, 835–838.

5. Stierle, A.; Keller, T. F.; Noei, H.; Vonk, V.; Roehlsberger, R. DESY NanoLab. J. Large-Scale Res. Facil. 2016, 2, A76.

6. Schropp, A. et al. PtyNAMi: Ptychographic Nano-Analytical Microscope. J. Appl. Crystallogr. 2020, 53, 957-971.

7. Schroer, C. G. et al. Nanofocusing Parabolic Refractive X-ray Lenses. Appl. Phys. Lett. 2003, 82, 1485.

8. Schroer, C. G. et al. Hard X-ray Nanoprobe Based on Refractive X-ray Lenses. Appl. Phys. Lett. 2005, 87, 124103.

9. Björling, A. et al. Ptychographic characterization of a coherent nanofocused X-ray beam. Optics express 28(4) 5069-5076 (2020).

10. Leake, S. J. et al. The Nanodiffraction beamline ID01/ESRF: a microscope for imaging strain and structure. Journal of synchrotron radiation, 2019, 26(2), 571-584.

11. Villar, F. et al. Nanopositioning for the ESRF ID16A nano-imaging beamline. Synchrotron Radiation News, 2018, 31(5), 9-14.

12. Winarski, R. P. et al. A hard X-ray nanoprobe beamline for nanoscale microscopy. Journal of Synchrotron Radiation 2012, 19(6), 1056-1060.

13. Benecke, G. et al. A Customizable Software for Fast Reduction and Analysis of Large X-ray Scattering Data Sets: Applications of the New DPDAK Package to Small Angle X-ray Scattering and Grazing-Incidence Small Angle X-ray Scattering. J. Appl. Cryst. 2014, 47, 1797-1803.

14. Thibault, P. et al. High-Resolution Scanning X-ray Diffraction Microscopy. Science 2008, 321, 379-382.

15. Doolette, C. L. et al. Tandem Probe Analysis Mode for Synchrotron XFM: Doubling Throughput Capacity. Anal. Chem. 2022, 94(11), 4584-4593.

16. Chayanun, L. et al. Combining Nanofocused X-Rays with Electrical Measurements at the NanoMAX Beamline. Crystals 2019, 9, 432.

17. Sow, C. et al. Unraveling the Spatial Distribution of Catalytic Non-Cubic Au Phases in a Bipyramidal Microcrystallite by X-ray Diffraction Microscopy. ACS Nano 2020, 14, 9456-9465.

18. Doolette, C. L. et al. Tandem Probe Analysis Mode for Synchrotron XFM: Doubling Throughput Capacity. Anal. Chem. 2022, 94(11), 4584.
